# Involvement of *ANXA5* and *ILKAP* in Susceptibility to Malignant Melanoma

**DOI:** 10.1371/journal.pone.0095522

**Published:** 2014-04-17

**Authors:** Yoana Arroyo-Berdugo, Santos Alonso, Gloría Ribas, Maider Ibarrola-Villava, María Peña-Chilet, Conrado Martínez-Cadenas, Jesús Gardeazabal, Juan Antonio Ratón-Nieto, Ana Sánchez-Díez, Jesús María Careaga, Gorka Pérez-Yarza, Gregorio Carretero, Manuel Martín-González, Cristina Gómez-Fernández, Eduardo Nagore, Aintzane Asumendi, María Dolores Boyano

**Affiliations:** 1 Department of Cell Biology and Histology, School of Medicine and Dentistry, University of the Basque Country (UPV/EHU), Leioa, Bizkaia, Spain; 2 Department of Genetics, Physical Anthropology and Animal Physiology, Faculty of Science and Technology, University of the Basque Country (UPV/EHU), Leioa, Bizkaia, Spain; 3 Department of Hematology and Medical Oncology, Instituto Investigación Sanitaria, INCLIVA, Valencia, Spain; 4 Department of Medicine, University of Castellon Jaume I, Castellon, Spain; 5 Department of Dermatology, Ophthalmology and Otorhinolaryngology, UPV/EHU, Service of Dermatology, BioCruces Health Research Institute, Cruces University Hospital, Barakaldo, Bizkaia, Spain; 6 Department of Dermatology, Ophthalmology and Otorhinolaryngology, UPV/EHU, Service of Dermatology, BioCruces Health Research Institute, Basurto University Hospital, Bilbao, Bizkaia, Spain; 7 Department of Dermatology, Doctor Negrin Hospital, Las Palmas de Gran Canaria, Spain; 8 Department of Dermatology, Ramón y Cajal Hospital, Madrid, Spain; 9 Deparment of Dermatology, La Paz University Hospital, Madrid, Spain; 10 Department of Dermatology, Instituto Valenciano de Oncología, Valencia, Spain; National Cancer Center, Japan

## Abstract

Single nucleotide-polymorphisms (SNPs) are a source of diversity among human population, which may be responsible for the different individual susceptibility to diseases and/or response to drugs, among other phenotypic traits. Several low penetrance susceptibility genes associated with malignant melanoma (MM) have been described, including genes related to pigmentation, DNA damage repair and oxidative stress pathways. In the present work, we conducted a candidate gene association study based on proteins and genes whose expression we had detected altered in melanoma cell lines as compared to normal melanocytes. The result was the selection of 88 *loci* and 384 SNPs, of which 314 fulfilled our quality criteria for a case-control association study. The SNP rs6854854 in *ANXA5* was statistically significant after conservative Bonferroni correction when 464 melanoma patients and 400 controls were analyzed in a discovery Phase I. However, this finding could not be replicated in the validation phase, perhaps because the minor allele frequency of SNP rs6854854 varies depending on the geographical region considered. Additionally, a second SNP (rs6431588) located on *ILKAP* was found to be associated with melanoma after considering a combined set of 1,883 MM cases and 1,358 disease-free controls. The OR was 1.29 (95% CI 1.12–1.48; p-*value* = 4×10^−4^). Both SNPs, rs6854854 in *ANXA5* and rs6431588 in *ILKAP*, show population structure, which, assuming that the Spanish population is not significantly structured, suggests a role of these *loci* on a specific genetic adaptation to different environmental conditions. Furthermore, the biological relevance of these genes in MM is supported by *in vitro* experiments, which show a decrease in the transcription levels of *ANXA5* and *ILKAP* in melanoma cells compared to normal melanocytes.

## Introduction

Malignant melanoma (MM) is a stepwise tumor process in which normal melanocytes in the basal layer of the epidermis acquire genetic aberrations that drive progression to melanoma. In recent years, there has been a constant increase in the incidence of MM among the world population, which is a worrying fact because MM is a highly aggressive, potentially lethal form of cancer [1]. Summaries of national melanoma notifications provided to the International Agency for Research on Cancer (IARC) (2002) demonstrate that the highest reported national incidence rates for melanoma occurred in the populations of Australia (39∶100,000 per year) and New Zealand (34∶100,000 per year). The next highest national melanoma rates were observed in the USA (17∶100,000 per year) followed by the countries in northern (Denmark, Norway and Sweden) and western European countries (the Netherlands and the United Kingdom), with incidences of 9–15∶100,000 per year [2], [3]. According to data published by the Spanish National Epidemiology Carlos III Health Institute, the annual incidence of melanoma in Spain is close to the adjusted annual incidence for the European population of 6.14 per 100,000 population in men and 7.26 per 100,000 population in women [3]. The predominantly non-Caucasian populations of Africa and Asia reported melanoma rates less than 3∶100,000 per year [2]. Likewise, MM tends to occur more often in people with light skin, hair and eyes, who seem to be more sensitive to the sun’s ultraviolet radiation, although the development of this pathology may happen in any geographical human group [4].

In this regard, epidemiological and genetic studies show that both the development of the disease and its evolution are determined by individual-specific genetic factors, genetic and epigenetic aberrations acquired by the tumor, and by environmental conditions [5], [6], [7]. Two high-risk melanoma susceptibility genes with large effects but low frequency in the population have been described. The best-established high-risk *loci* for melanoma susceptibility are the genes *CDKN2A,* located on chromosome 9p21, and *CDK4* on 12q14. The *CDKN2A* locus encodes for both proteins p16^INK4a^ and p14^ARF^ and accounts for susceptibility in 25–40% of melanoma families [8], [9]. Mutations in *CDK4* are rare and worldwide only three families have been reported to carry mutations on this gene worldwide [10], [11]. However, familial melanoma only comprises approximately 10% of all MM cases, so it seems likely that there are other low-penetrance polymorphisms with small effects but very common in the population also associated with the susceptibility to develop MM. The *MC1R* gene is the candidate locus *par excellence* associated with the appearance of different pigmentary phenotypes as well as to the ability to modulate the susceptibility to develop sporadic MM [12], [13]. However, other these low-penetrance alleles have been reported which belong to biological processes such as pigmentation (*ASIP*, *OCA2*, *SLC45A2, TYR*, *TYRP1)*, immune response (*IL-1β*, *IL-10*, *INF-γ*, *TNF-α*), DNA repair (*ERCC1*, *ERCC2*, *MGMT*, *TERT1*, *TRF1*, *XRCC1*, *XRCC3*), and metabolism (*GSTM1*, *GSTP1*, *GSTT1*), including the vitamin D receptor [12], [14]–[19].

Despite the fact that investigating the molecular alterations involved in the pathogenesis of MM is a topic of active research, the advances achieved so far are still insufficient to establish a set of biomarkers and molecular targets which will facilitate an early diagnosis, predicting the risk for metastasis in melanoma patients, and developing more efficient therapies against this neoplasm. In this context, we have conducted a case-control association study based on 384 SNPs distributed in 88 candidate genes, of which 314 were successfully genotyped. These SNPs were selected by the combination of the information obtained from proteomic analysis by two-dimensional electrophoresis and mass spectrometry, and mRNA expression arrays performed previously by our group. The selected SNPs were genotyped in a case-control study using three series of Spanish population samples (1,883 MM patients and 1,358 disease-free controls in total). Our results showed that the SNPs rs6854854, located in *ANXA5* and, rs6431588 in *ILKAP* are associated with MM.

## Results

The candidate genes to be genotyped in the present work were selected based on proteins and genes differentially expressed between melanoma cell lines and primary melanocytes. Therefore, previous proteomic analyses were performed on six melanoma cell lines (A375, Hs294T, HT-144, 1205Lu, WM793B, JSG) and four primary melanocytes (HEMn-LP, HEMn-MP, HEMn-DP, HEMa-LP) using two-dimensional electrophoresis (2D-PAGE), while Affymetrix Human U133A GeneChip arrays were used to analyze and compare mRNA expression profiles on four different melanoma cell lines isolated from patients’ biopsies in our laboratory (which were named JEM, JSG1, JSG2, MJOI) and three benign nevi from patients’ skin lesions isolated also in our laboratory (named FDR, JPA, RRR) (unpublished data). Finally, we selected 88 genes involved in cell growth, cell cycle and apoptosis, cell signaling, transcription and stress response (Table 1). A total of 384 SNPs were selected on the basis of their Linkage Disequilibrium (LD) profiles using Haploview [20].

**Table 1:** Eighty-eight candidate genes selected for SNPs genotyping.

Category	Genes
Cell growth, cellcycle and apoptosis	*ANXA5, APC, AR, AURKB, AXL, BAX, BCL2L11, CDK2, CDK4, CDKN1A,* *CTSD, ENO1, FASTK, GAS6, GSTP1, HDGF, IGFBP5, ILKAP, IMPDH2,* *MAGED1, MAPRE1, NDN, NME2, NPM1, NRAS, PEA15, PIM1, PSMB3,* *PSMD11, RAD50, RAP1B, RBL2, RCC2, RELA, SPRR2G, TGFB1, WEE1*
Cell signaling	*AR, BAG2, CALM3, CCT7, CLIC1, CSNK1G2, FSCN1, GDI2, GRB2, ITGA5,* *ITGAM, MAPKAPK3, MCL1, MYD88, PDPK1, PEBP1, PKM2, RAC1,* *RCN1, RPSA, SIAH2, SNAI1, THBS1, TPM2, TTC1, UBE2L6, WARS, WNT5A, YWHAZ*
Transcription	*ARID5A, CREBBP, CTBP1, CTNNB1, EEF1D, FOS, HDAC5,* *HTATIP, JUN, PIR, RUVBL1, RUVBL2, SP1, TFDP1, PIAS3*
Stress response	*DUSP1, GPX1, PARK7, PRDX1, PRDX3, SOD2, TXNL1*

In discovery Phase I, a total of 464 patients with MM and 400 volunteer cancer-free controls were genotyped. From the initial list of 384 SNPs, 70 SNPs were discarded in Phase I for the following reasons: 17 SNPs because they could be genotyped in less than 85% of the samples; 5 SNPs due to a low quality genotyping; 18 SNPs were found to be monomorphic in our control population and patients; and 30 SNPs were out of Hardy-Weinberg equilibrium (HWE) after a conservative Bonferroni correction for multiple testing. The list of removed SNPs is provided in Table S1 in File S1.

### Comparison of Allele Frequencies

**Figure 1:**
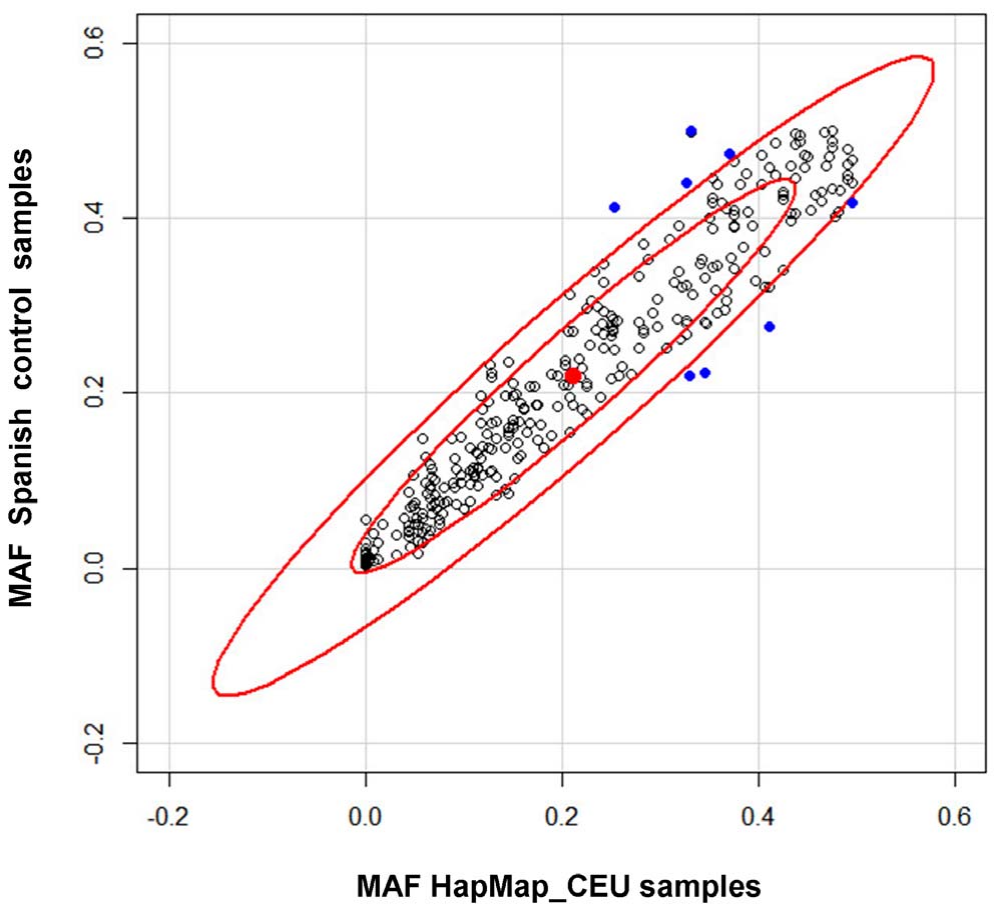
Comparison of minor allele frequencies (MAF), Spanish *vs.* HapMap European data. The small and big red circles show the range within one and two standard deviation (SD) of the mean, respectively. Blue dots represent values that significantly differ from HapMap European data.

Therefore, the study continued with 314 SNPs (Table S2 in File S1), whose allele frequencies were estimated based on our control samples and compared to those of the HapMap CEU population (people with Northern and Western European ancestry). Frequencies were very similar and a high positive correlation can be observed in Figure 1 (R^2^ = 0.92), suggesting that our study population is sufficiently homogeneous to conduct genetic association studies with minor risk of population stratification. However, one-sample *t*-test gave evidence that the corresponding Spanish minor allele frequency (MAF) differed from that published in HapMap (p-*value* <0.05) for 8 of the 314 SNPs considered (rs2069502 in *CDK4*, rs3731239 in *CDKN1A*, rs2303942 in *FASTK*, rs2497 in *GDI2*, rs2088702 in *PEBP1*, rs228275 in *PSMB3*, rs17800727 in *RBL2*, rs6586542 in *RCC2*) (shown as blue dots in Figure 1).

### Associations with MM Risk

**Figure 2:**
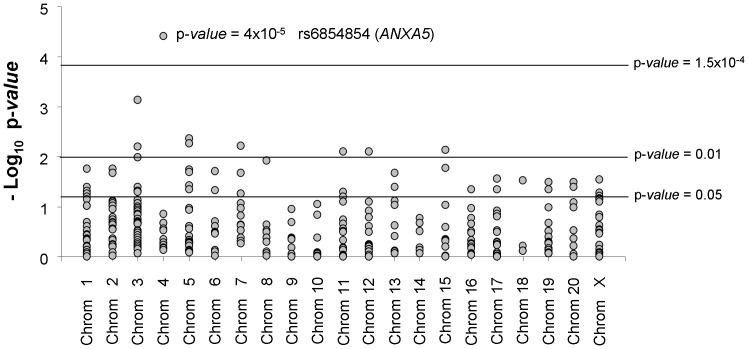
SNP association results. The –log10 of the allelic p-values from 314 SNPs comparing 464 melanoma patients and 400 controls of Spanish origin at Phase I are represented. The chromosomal SNP distribution is shown. The SNP rs6854854 in *ANXA5* remained statistically significant after Bonferroni correction (p-*value* <0.00015).

After a Fisher’s exact test to compare allele counts between cases and controls, 38 SNPs located in 31 genes were associated with MM in the Spanish population considering a p-*value* threshold of 0.05. Representation of –log10 p-*values* for the comparison of minor allele frequency (MAF) between the 464 MM cases and the 400 controls are detailed in Figure 2. Detailed information on SNP, gene, chromosome location, MAF, odds ratio (OR), 95% confidence interval (95% CI) and p-*value* for these 37 SNPs are presented in Table 2. If a more restrictive p-*value* threshold of 0.01 is established, 9 SNPs remain as candidates associated with MM in our Spanish population. And among them, only rs6854854, located in the intron 2 of *ANXA5,* showed an association that was statistically significant (p-*value* = 4×10^−5^), after applying the Bonferroni correction for multiple comparisons. This means that individuals who possess at least one *ANXA5* rs6854854 C allele are protected against developing MM relative to those with the reference genotype (OR = 0.541; 95% CI = 0.371–0.791; p-*value* = 0.0013). The *ANXA5* rs6854854 locus was significant under the additive (p-*value = *9.06×10^−5^) and recessive genetic models (p*-value = *5.97×10^−5^).

**Table 2:** SNPs associated with Malignant Melanoma p<0.05 in Spanish population analyzed in Phase I.

Gene	SNP	Chrom	Controls MA	Controls MAF	Cases MAF	Fisherp-*value*	OR (95% CI)	p-*value*
*ANXA5*	rs6854854	4	C	0.115	0.059	0.00004	0.541 (0.371–0.791)	0.0013
*CTNNB1*	rs4135385	3	G	0.273	0.202	0.0007	0.652 (0.494–0.862)	0.0025
*APC*	rs13167522	5	C	0.125	0.083	0.0043	0.638 (0.451–0.969)	0.0040
*TTC1*	rs6556466	5	C	0.094	0.058	0.0054	0.645 (0.434–0.957)	0.0280
*FASTK*	rs2288648	7	A	0.008	0	0.0061	0.061 (0.003–1.081)	0.0060
*SIAH2*	rs8072	3	T	0.013	0.002	0.0063	0.173 (0.037–0.806)	0.0110
*NDN*	rs1722807	15	A	0.010	0	0.0074	0.060 (0.003–1.074)	0.0059
*WEE1*	rs11042431	11	G	0.211	0.160	0.0079	0.817 (0.611–1.092)	0.1710
*ITGA5*	rs12318746	12	A	0.010	0	0.0079	0.061 (0.003–1.095)	0.0064
*RUVBL1*	rs11719889	3	A	0.219	0.275	0.0102	1.425 (1.079–1.883)	0.0120
*EEF1D*	rs4874163	8	G	0.155	0.113	0.0121	0.697 (0.525–0.926)	0.0120
*PKM2*	rs2959910	15	C	0.009	0	0.0172	0.072 (0.004–1.305)	0.0123
*CCT7*	rs2231427	2	G	0.009	0	0.0174	0.072 (0.004–1.312)	0.0125
*ENO1*	rs11544514	1	A	0.008	0	0.0179	0.073 (0.004–1.326)	0.0129
*APC*	rs4987109	5	C	0.009	0	0.0180	0.073 (0.004–1.326)	0.0130
*BAG2*	rs9370567	6	C	0.093	0.062	0.0198	0.698 (0.472–1.033)	0.0710
*RAD50*	rs4526098	5	G	0.029	0.011	0.0205	0.453 (0.213–0.964)	0.0354
*RAC1*	rs6951997	7	G	0.040	0.019	0.0210	0.506 (0.270–0.948)	0.0305
*ILKAP*	rs6431588	2	T	0.146	0.188	0.0214	1.358 (1.049–1.768)	0.0210
*GAS6*	rs7997328	13	C	0.268	0.320	0.0216	1.427 (1.084–1.877)	0.0110
*PSMD11*	rs7212835	17	G	0.165	0.127	0.0278	0.739 (0.561–0.968)	0.0270
*PIR*	rs1996173	X	T	0.016	0	0.0290	0.053 (0.003–0.933)	0.0032
*TXNL1*	rs655539	18	C	0.105	0.075	0.0294	0.730 (0.507–1.050)	0.0880
*TGFB1*	rs2241715	19	T	0.370	0.320	0.0317	0.792 (0.601–1.045)	0.0990
*MAPRE1*	rs2235760	20	T	0.148	0.113	0.0326	0.714 (0.520–0.982)	0.0370
*APC*	rs1882619	5	C	0.083	0.057	0.0377	0.716 (0.478–1.074)	0.1050
*GAS6*	rs6602910	13	G	0.389	0.439	0.0399	1.360 (1.021–1.811)	0.0350
*MCL1*	rs12036617	1	T	0.006	0	0.0400	0.089 (0.005–1.665)	0.0261
*MAPRE1*	rs242553	20	T	0.497	0.547	0.0401	0.767 (0.566–1.040)	0.0870
*MYD88*	rs989298	3	A	0.006	0	0.0411	0.090 (0.005–1.684)	0.0270
*RBL2*	rs17800727	16	G	0.413	0.365	0.0445	0.980 (0.740–1.297)	0.8870
*TTC1*	rs3733868	5	T	0.068	0.045	0.0449	0.632 (0.407–0.980)	0.0390
*TGFB1*	rs8110090	19	G	0.068	0.046	0.0457	0.716 (0.461–1.111)	0.1350
*GRB2*	rs7219	17	G	0.270	0.228	0.0460	0.814 (0.617–1.073)	0.1430
*BAG2*	rs9885757	6	T	0.270	0.228	0.0465	0.782 (0.594–1.029)	0.0790
*MAPKAPK3*	rs11130254	3	G	0.148	0.115	0.0480	0.769 (0.557–1.063)	0.1110
*RCC2*	rs1204897	1	A	0.207	0.170	0.0486	0.763 (0.573–1.016)	0.0630

Chrom. Chromosome; MA. Minor Allele; MAF. Minor Allele Frequency; OR (95% CI). Odds ratio (95% confidence interval).

From these 38 SNPs with a p-value <0.05, we selected 15 SNPs with a MAF greater than 0.05 in our control samples for validation in Phase II, which consisted on an independent set of 507 MM cases and 383 controls. None of the SNPs presented a statistically significant association with MM at this stage (Table 3). However, four of them had an overall p-*value* <0.05 when phases I and II were considered together (971 MM cases and 783 controls). These four SNPs were: rs13167522 in *APC* (p-*value* = 0.0035); rs4874163 in *EEF1D* (p-*value* = 0.0077); rs6431588 in *ILKAP* (p-*value* = 0.0086); and rs7212835 in *PSMD11* (p-*value* = 0.038). As association studies require large numbers of samples to detect weak association and increasing study size typically has a large effect on power, these four SNPs were genotyped in 912 new MM cases and 581 controls in validation Phase III.

**Table 3:** Data of the SNPs analyzed in Phase II.

Gene	SNP	Chrom	Phase IIFisherp-*value*	Phase IIOR(95% CI)	Phase IIp-*value*	Phase I+IIOR(95% CI)	Phase I+IIp-*value*
*GAS6*	rs7997328	13	0.217	1.253(0.959–1.636)	0.097	1.336(1.103–1.617)	0.003
*ILKAP*	rs6431588	2	0.157	1.198(0.935–1.535)	0.157	1.322(1.076–1.623)	0.007
*APC*	rs13167522	5	0.236	0.813(0.583–1.145)	0.236	0.730(0.569–0.937)	0.013
*EEF1D*	rs4874163	8	0.201	0.831(0.630–1.101)	0.201	0.757(0.607–0.944)	0.013
*CTNNB1*	rs4135385	3	0.941	0.949(0.721–1.250)	0.711	0.788(0.648–0.958)	0.016
*TTC1*	rs3733868	5	0.315	0.744(0.478–1.158)	0.189	0.685(0.501–0.935)	0.016
*ANXA5*	rs6854854	4	0.579	1.122(0.775–1.623)	0.542	0.787(0.606–1.022)	0.071
*PSMD11*	rs7212835	17	0.501	0.907(0.687–1.202)	0.501	0.823(0.663–1.022)	0.077
*RUVBL1*	rs11719889	3	0.901	0.972(0.743–1.272)	0.837	1.169(0.964–1.418)	0.112
*TTC1*	rs6556466	5	0.881	1.078(0.732–1.586)	0.704	0.839(0.638–1.105)	0.211
*TXNL1*	rs655539	18	0.881	1.078(0.732–1.586)	0.704	0.874(0.672–1.137)	0.315
*WEE1*	rs11042431	11	0.914	1.011(0.761–1.343)	0.941	0.911(0.744–1.116)	0.367
*TGFB1*	rs2241715	19	0.724	1.082(0.827–1.417)	0.565	0.929(0.766–1.126)	0.452
*RBL2*	rs17800727	16	0.524	1.126(0.851–1.490)	0.407	1.047(0.859–1.277)	0.647
*APC*	rs1882619	5	0.297	1.242(0.820–1.881)	0.305	0.939(0.704–1.252)	0.667

Chrom. Chromosome; OR (95% CI) Odds ratio (95% confidence interval).

**Figure 3:**
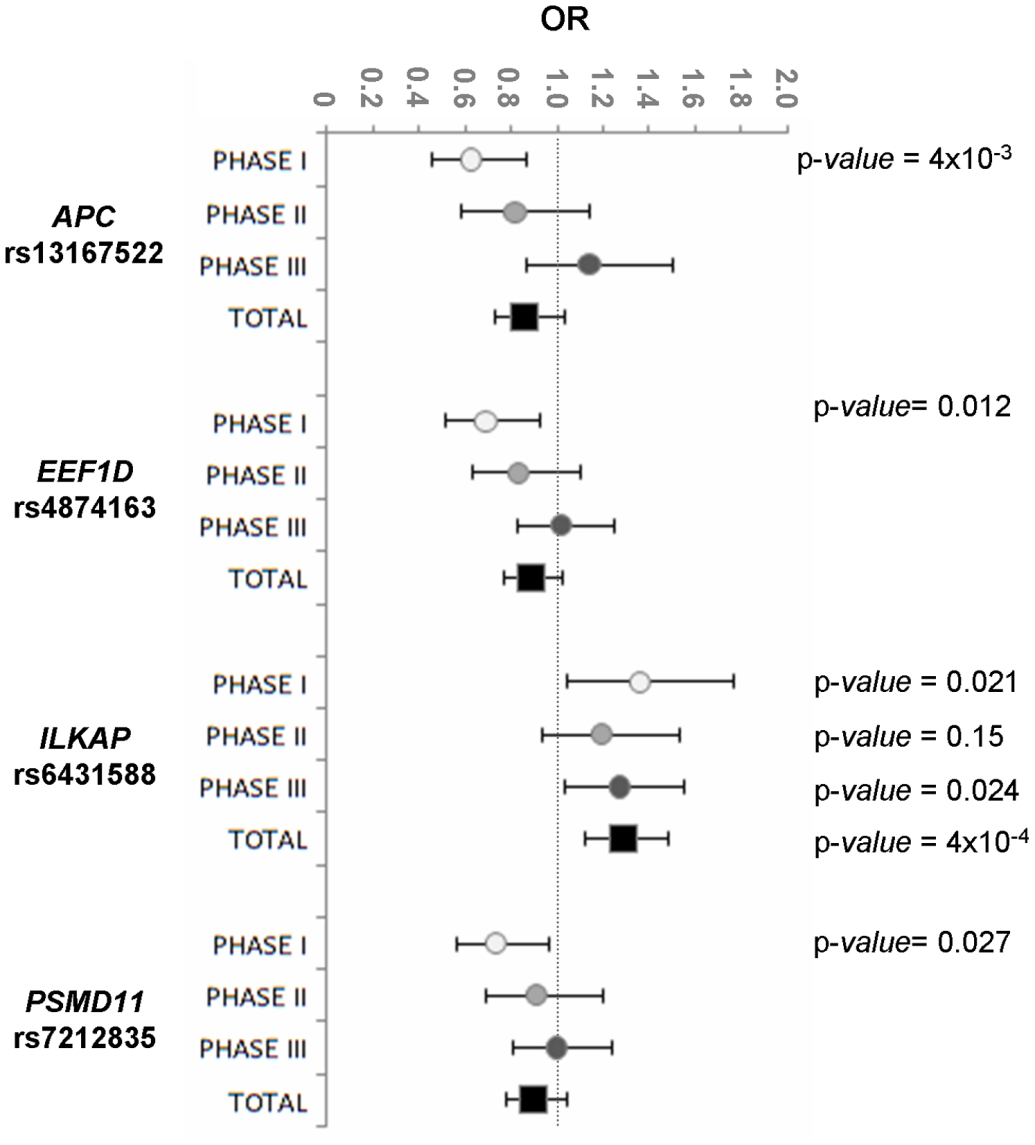
Forest plot showing the odds ratios and 95% confident intervals for the four SNPs most associated with melanoma risk predisposition. Dots represent odds ratios for each phase, discovery (Phase I) and validations (Phases II and III). The non-shown p-*values* are not statistically significant.

After genotyping 1,883 patients with melanoma and 1,358 control subjects, one SNP in intron 3 of *ILKAP* gene (rs6431588) was associated with increased risk of developing MM in the Spanish population (Fisher’s test p-*value* = 5×10^−4^). The OR was 1.29 (95% CI 1.12–1.48; p-*value* = 4×10^−4^) (Figure 3). Therefore, the *ILKAP* rs6431588 locus was significant under the additive genetic model (p*-value = *5.8×10^−4^, using the Cochrane-Armitage trend test). For the other three remaining SNPs p-*values* were not statistically significant.

### *ANXA5* and *ILKAP* Expression in Human Melanoma Cell Lines and Primary Melanocytes

**Figure 4:**
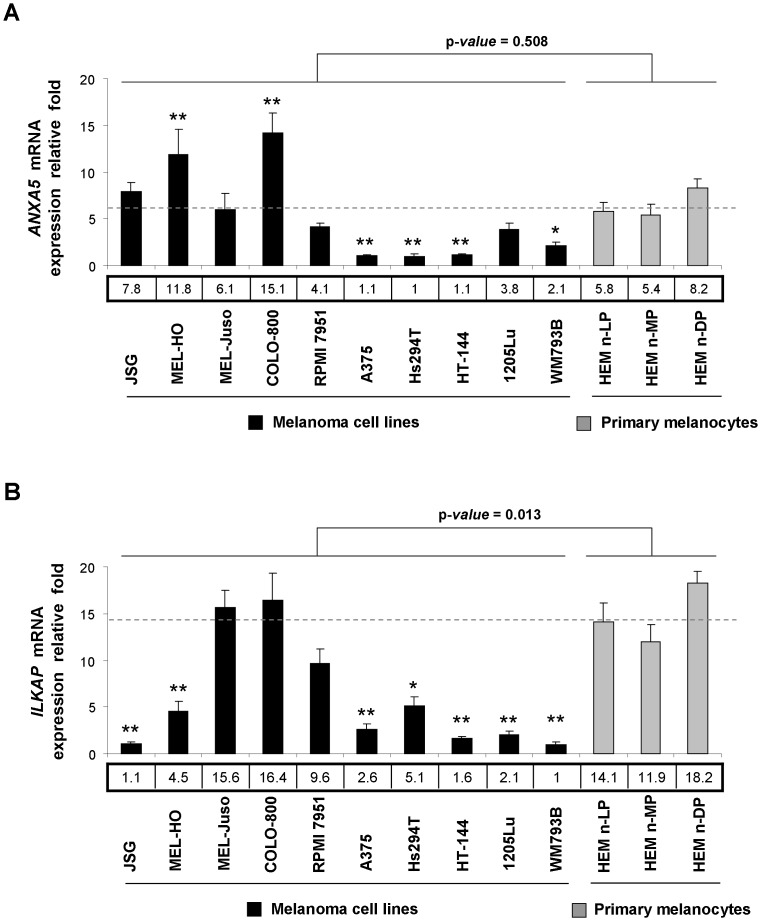
*ANXA5* and *ILKAP* mRNA expression in human melanoma cell lines and primary melanocytes. A) *ANXA5* relative expression levels. B) *ILKAP* relative expression levels. Results are expressed as mean relative expression fold ± standard deviation (SD) in the histograms, calculated on six replicates of each sample. The relative expression fold values are reported in boxes below histograms. The average expression of primary melanocytes is shown with a dashed line. Statistically significant differences of each melanoma cell line with respect to primary melanocytes: * p-*value* ≤0.05; ** p-*value* ≤0.01.

**Figure 5:**
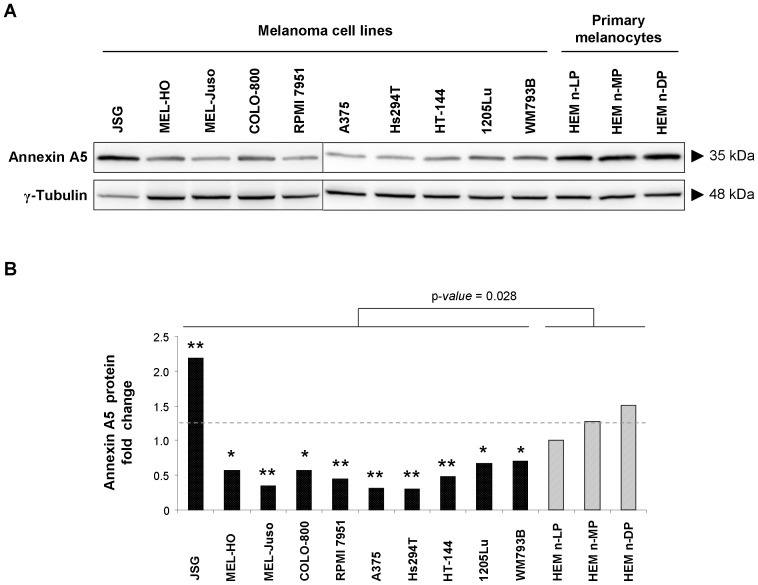
Western blot analysis for Annexin A5 in melanoma cell lines and primary melanocytes. (A) Western blotting shows a decrease in Annexin A5 protein levels in melanoma cell lines with respect to primary melanocytes; γ-tubulin was used as the internal control. (B) Histogram showing the relative expression levels of Annexin A5 in melanoma cell lines and primary melanocytes.

In order to confirm an alteration in the expression levels of *ANXA5* and *ILKAP* in MM, quantitative measurement of these genes’ expression was investigated in 10 melanoma cell lines and 3 primary melanocytes using quantitative real time PCR (Figure 4). The results showed a weak reduction in the *ANXA5* gene expression and a significant decreased gene expression of *ILKAP* (p-*value* <0.05 after applying Student’s *t*-test) in the melanoma cell lines studied in comparison with primary melanocytes. Although some individual heterogeneity was also observed as not all the melanoma cell lines showed a reduction in *ANXA5* or *ILKAP* gene expression, the trend observed coincided with that obtained previously using expression microarrays, in which only *ILKAP*, but not *ANXA5*, showed significant differences at mRNA transcriptional levels between melanocytes and the tumor cell lines studied (unpublished data). Additionally, the results obtained from previous 2D-PAGE assays made in our laboratory suggested a decrease of both proteins ILKAP and Annexin A5 in melanoma cell lines compared to primary melanocytes. A Western blot confirmed a significant decrease of Annexin A5 protein amount in melanoma cell lines (Figure 5). These results suggest a deregulation of ILKAP at the transcriptional level, whereas the deregulation seems to be at posttranslational level for Annexin A5.

### *ILKAP* Coding and Promoter Region Sequence Analysis

As a change in the nucleotide sequence within the gene coding region can alter the expression and/or mRNA stability and thus the final protein concentration, we sequenced a cDNA fragment of 1,259 bp covering the whole *ILKAP* exons (Chr 2∶239,079,043–239,112,324 according to UCSC Genome Browser, GRCh37/hg19), in order to assess if there were nucleotide changes in the melanoma cell lines. However, the chromatograms showed no changes in the nucleotide sequence of the *ILKAP*.

In the absence of coding region mutations that could be associated to the *ILKAP* expression deregulation observed in melanoma cell lines, we hypothesized that there could exist mutations in the promoter region that could be altering the transcriptional activity of *ILKAP*. Therefore, the *ILKAP* gene promoter region was amplified from genomic DNA using two pairs of primers that resulted in one fragment of 1,443 bp and another overlapping fragment of 1,110 bp. The sequences obtained for each sample were aligned and assembled, enabling the reading of a sequence of 2,069 bp (Chr 2∶239,112,648–239,114,717). Twelve single nucleotide variants were found, all of them corresponding to already polymorphisms described. Table 4 shows the SNPs identified in the promoter region of *ILKAP* and the genotypes that each cell line presents. The 12 polymorphisms observed in the tested cell lines clustered into three different haplotypes, which are sorted from the position Chr 2∶239,114,642 to the position Chr 2∶239,113,023. The melanoma lines A375, HT-144 and JSG are homozygous for Haplotype 1 (TACCGGATCCGA). The cell lines 1205Lu, WM793B and HEMn-MP are homozygous for Haplotype 2 (CGTGGCGCCAAG). Finally, the cell lines Hs294T, HEMn-LP and HEMn-DP are homozygous for Haplotype 3 (CGTGACGCTAAG).

**Table 4:** Polymorphisms and alleles identified in the promoter region of *ILKAP* gene in each cell line studied.

		Melanoma cell lines						Primary melanocytes		
	Chr2 position	A375	Hs294T	HT-144	1205Lu	WM793B	JSG	HEMn-LP	HEMn-MP	HEMn-DP
**^1^rs13007964**	239,114,642	**T/T**	C/C	**T/T**	C/C	C/C	**T/T**	C/C	C/C	C/C
**^2^rs13006295**	239,114,577	A/A	**G/G**	A/A	**G/G**	**G/G**	A/A	**G/G**	**G/G**	**G/G**
**^3^rs13033116**	239,114,240	**C/C**	T/T	**C/C**	T/T	T/T	**C/C**	T/T	T/T	T/T
**^4^rs13000470**	239,114,218	**C/C**	G/G	**C/C**	G/G	G/G	**C/C**	G/G	G/G	G/G
**^5^rs11694064**	239,113,971	G/G	**A/A**	G/G	G/G	G/G	G/G	**A/A**	G/G	**A/A**
**^6^rs13001461**	239,113,961	**G/G**	C/C	**G/G**	C/C	C/C	**G/G**	C/C	C/C	C/C
**^7^rs34795319**	239,113,875	A/A	**G/G**	A/A	**G/G**	**G/G**	A/A	**G/G**	G/G	**G/G**
**^8^rs35519451**	239,113,863	**T/T**	C/C	**T/T**	C/C	C/C	**T/T**	C/C	C/C	C/C
**^9^rs11695186**	239,113,794	C/C	**T/T**	C/C	C/C	C/C	C/C	**T/T**	C/C	**T/T**
**^10^rs34272954**	239,113,510	C/C	**A/A**	C/C	**A/A**	**A/A**	C/C	**A/A**	**A/A**	**A/A**
**^11^rs13020362**	239,113,103	G/G	**A/A**	G/G	**A/A**	**A/A**	G/G	**A/A**	**A/A**	**A/A**
**^12^rs34193006**	239,113,023	A/A	**G/G**	A/A	**G/G**	**G/G**	A/A	**G/G**	**G/G**	**G/G**

The SNPs are numbered according to their appearance in the promoter region of *ILKAP* from the position Chr 2∶239,114,642 to the position Chr 2∶239,113,023 according to NCBI dbSNP 138. The ancestral allele is in black and the derived allele in bold.

### Tajima’s *D* Test Analysis to Identify Putative Signatures of Positive Selection

Due to the high frequency of polymorphisms observed in the promoter region of *ILKAP*, we decided to use the Tajima’s *D* test to investigate if this locus was subjected to the effect of Natural Selection in order to add evolutionary relevance to the function of this locus. Thus, we used data from 1000 Genomes Project to analyze a final region of 66 Kb (chr2∶239066043-239132324 according to UCSC Genome Browser website, GRCh37/hg19), which included the promoter and the coding region of *ILKAP*. The African population (n = 246) did not show a statistically significant value of Tajima’s *D* (*D* = 0.7; p-*value* = 0.16). However, the values of Tajima’s *D* obtained for Europeans (*D* = 2.54; p-*value* = 0.010; n = 380) and for the Asian population (*D* = 2.50; p-*value* = 0.013; n = 286) were statistically significant. These large and positive Tajima’s *D* test in *ILKAP* suggests that balancing selection could be playing a role on the evolutionary history of this locus.

## Discussion

Instead of selecting among candidate genes already described in the literature, the present work started by searching for genes whose expression (both at the protein and mRNA levels) is altered in melanoma cell lines as compared to normal melanocytes. After identifying the candidate genes in this way, a set of SNPs was selected for each corresponding gene. The result was the selection of 88 *loci* and 384 SNPs, of which 314 fulfilled our quality criteria.

The allele frequencies observed in our control samples were highly correlated to the HapMap CEU population (R^2^ = 0.92). Therefore, our experimental results are in good agreement with the recent report by Gayán *et al.* showing that the Spanish population is similar to Western and Northern Europeans and sufficiently homogeneous to conduct genetic association studies with minor risk of population stratification [21]. However, we also saw that in our control population the MAF for 8 of the 314 SNPs considered differed from that published in HapMap. This may be due to a role of these SNPs (or of really linked SNPs) in adaptive differences to environmental conditions because the frequency of these 8 SNPs seems to differ across populations with different geographic location (Table S3 in File S1).

From discovery Phase I, 38 SNPs showed p-*values* below 0.05 and, of these, only the *ANXA5* rs6854854 SNP remained statistically significant after Bonferroni correction. The annexins are a super-family of closely related calcium and membrane binding proteins which show cell type specific expression. Twelve annexins, named as annexins A1–A11 and A13, have been described common to vertebrates [22], [23]. This protein family has a wide variety of cellular functions including vesicle trafficking, cell division, apoptosis, calcium signaling and growth regulation [24], [25]. Although Annexin A5 was the first annexin characterized for three-dimensional structure in 1990 [26], its exact physiological function has not been fully understood. Recent data suggest that the invasion capacity, a main characteristic of tumors, is at least in part regulated by Annexin A5 in different cancer types [27], [28], [29]. Thus, Wehder *et al.* (2009) detected a decreased migration activity and invasion capability of head and neck squamous cell carcinoma after lacking *ANXA5* [29]. Therefore, the reduction of Annexin A5 protein amount observed in melanoma cell lines may be changing motile capacity of the tumor cells. However, it should be aware that *ANXA5* seems to have specific effects on distinct types of tumors [27], so experimentally functional analysis are needed to determine the specific role of Annexin A5 in MM.

Thereby, although the results obtained suggest that allele C in rs6854854 has a protective role against melanoma (p-*value* = 4×10^−5^), with an OR of 0.541 (95% CI 0.371–0.791; p-*value* = 0.0013), after genotyping 464 MM patients and 400 disease-free controls, the *ANXA5* rs6854854 SNP did not reach statistical significance in validation Phase II. It is possible that the difficulty in replication of results may be due to the existence of a change in the minor C allele frequency in the control group of Phase II. The C allele frequency in HapMap for European population (CEU) is 0.110 which matches the frequency obtained for the control group in Phase I of the study, however, the control population tested in the Phase II showed a minor C allele frequency of 0.075. An analysis of allele frequencies in different populations using data from 1000 Genomes Project supports a possible population structure for rs6854854 (Table 5). These data suggest the need for a new validation in a population where C minor allele frequency remains similar to that described in HapMap for European population. On the other hand, although using HWE as a screening tool removed part of the SNPs selected for analysis, SNP rs17718 (out of HW equilibrium and located in the *ANXA5* 3′UTR) turned out to be significantly associated with MM. Fardo *et al.* (2009) found that true disease susceptibility loci subjected to various patterns of genotype miscalls can be largely out of HWE and, thus, be candidates for removal before association testing [30].

**Table 5:** Minor allele frequency for the SNPs found in the promoter region of *ILKAP* in different populations (Data from 1000 Genomes Phase I May 2011).

		Minor allele frequency					
SNP	*Gene*	Europe (n = 380)		Africa (n = 246)		Asia (n = 286)	
rs6854854	*ANXA5*	0.075	C	0.278	C	0	C
rs6431588	*ILKAP*	0.179	T	0.122	T	0.003	T
rs13007964	*ILKAP*	0.333	T	0.197	T	0.203	T
rs13006295	*ILKAP*	0.333	A	0.197	A	0.203	A
rs13033116	*ILKAP*	0.333	C	0.197	C	0.205	C
rs13000470	*ILKAP*	0.328	C	0.197	C	0.205	C
rs11694064	*ILKAP*	0.169	A	0.169	A	0.010	A
rs13001461	*ILKAP*	0.333	G	0.197	G	0.203	G
rs34795319	*ILKAP*	0.320	A	0.197	A	0.203	A
rs35519451	*ILKAP*	0.333	T	0.197	T	0.203	T
rs11695186	*ILKAP*	0.217	T	0.179	T	0.030	T
rs34272954	*ILKAP*	0.333	C	0.197	C	0.203	C
rs13020362	*ILKAP*	0.333	G	0.197	G	0.203	G
rs34193006	*ILKAP*	0.333	A	0.197	A	0.203	A

After genotypig a total of 1,883 MM cases and 1,358 controls, SNP rs6431588, located in *ILKAP*, was a new locus associated with a higher susceptibility to MM. Therefore, our data suggest that individuals who possess the T allele in the *ILKAP* rs6431588 locus are more likely to develop MM, fitting to an additive model of inheritance.

The 1000 Genomes database shows that the frequency of the T allele in rs6431588 varies significantly depending on the geographical region considered (Table 5). The minor allele of rs6431588 appears more frequently in the European populations than in the rest of geographic regions. In fact, it seems that rs6431588 has a population structure where the frequency of the T allele increases from Africa towards the North of Europe. Some findings support the hypothesis that latitudinal genetic diversity gradients are present in humans and reflect genetic adaptations to different environmental pressures that have shaped the human genome [31], [32], [33]. Latitude appears to provide a good proxy for the selective pressures that shaped variation in our genome because it is correlated with different variables like mean winter and summer temperatures, rainfall or ultraviolet radiation exposure, which appears to be the predominant environmental risk factor for MM.

We also have experimentally observed that the levels of *ILKAP* gene expression decrease in melanoma cells compared to normal melanocytes. In this regard, the ILKAP (Integrin-linked kinase-associated serine/threonine phosphatase 2C) plays a role in the regulation of diverse processes such as cell cycle progression, migration and cell death, and appears to have an important role in oncogenic transformation [34], [35], [36]. Thus, it is possible that decreasing *ILKAP* expression levels favor a constitutive activation of ILK (Integrin-linked kinase). In its turn, this activation inactivates instead GSK3β which favors the stabilization and nuclear translocation of β-catenin, which results in the subsequent activation of the TCF/LEF family of transcription factors that promote cell survival and proliferation [35]. In fact, a high activation of ILK is associated with poor outcome in patients with melanoma [37]. Likewise, low levels of *ILKAP* may reduce apoptosis induced by Tumor Necrosis Factor alpha (TNFα) and the presence of reactive oxygen species (ROS), as well as reduce the formation of complexes with RSK2 (Ribosomal protein S6 kinase-2) in the nucleus and consequently enhance the expression of Cyclin D1 (a RSK2 downstream substrate), which ultimately promotes tumor cell survival and proliferation [36]. Researchers have thus far considered ILKAP a cytoplasmic protein, however, its location also in the nucleus opens a window to unknown functions of ILKAP. Anyway, ILKAP and Annexin A5 functions are not the main focus of the present work and it would be necessary to perform experimentally functional analysis to determine whether the down-regulation of these genes has actually a role in MM.

**Figure 6:**
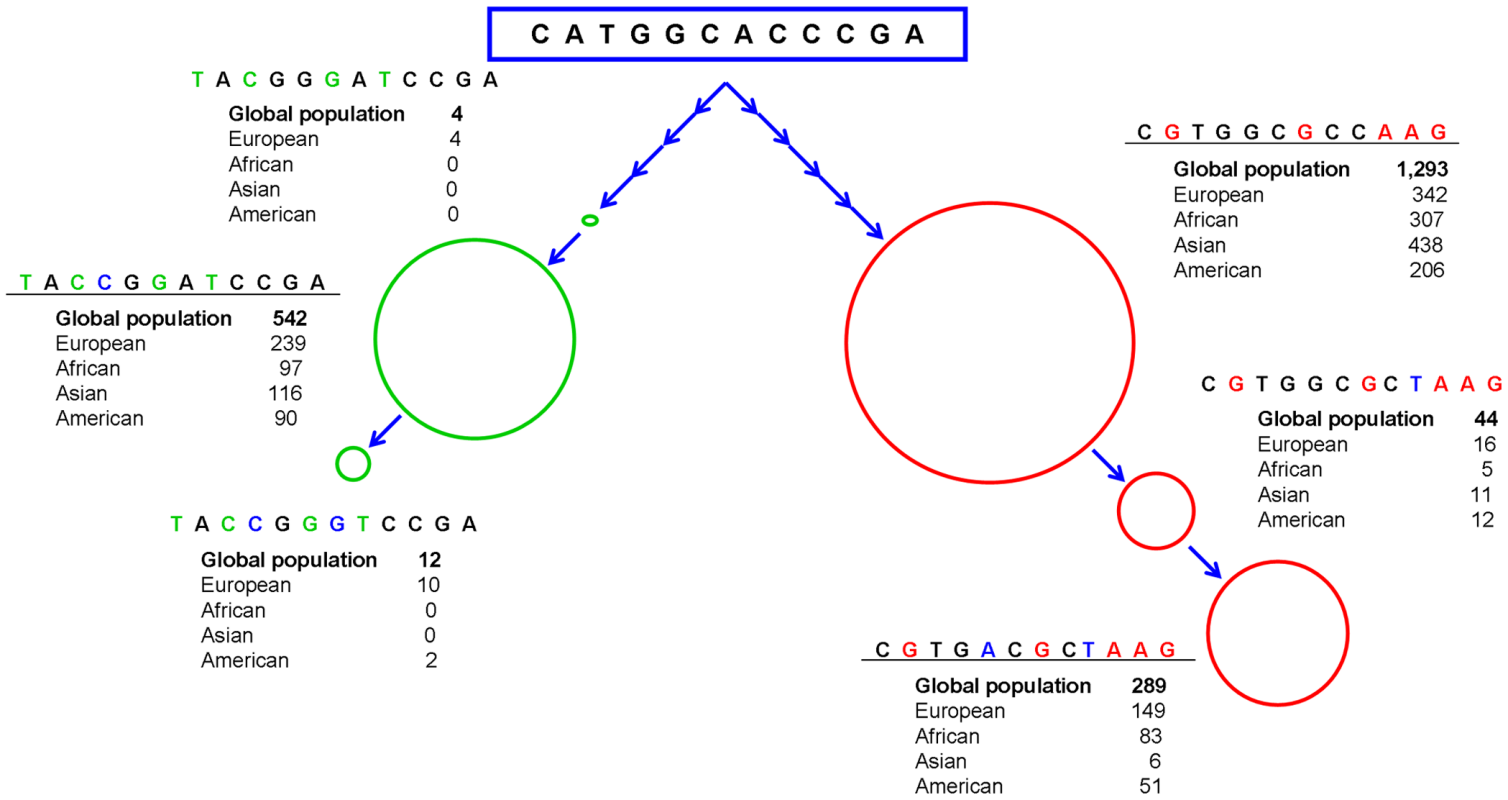
Haplotypes in the promoter region of *ILKAP* gene in different populations obtained from 1000 Genomes Project. Although we detected three different haplotypes among the melanoma cell lines and the primary melanocytes studied (underlined haplotypes), a total of six different haplotypes have been reported worldwide. Each haplotype is represented with a circle, whose size is proportional to their frequency in the global population. The genealogic relationship suggests the existence of two different main lineages, showed in green and red circles.

On the other hand, despite the emphasis put on functional analyses of coding SNPs, many SNPs are located in non-coding regulatory regions whose exact functions are not yet clear, but that could be influencing the binding affinity of transcription factors, and thus they could be exerting an important biological regulatory role [38]. As we detected no variation within the coding region, we decided to investigate the 5′ region of *ILKAP*, where we detected 12 SNPs. The minor allele frequencies for the 12 SNPs found in the *ILKAP* gene promoter region are very similar for all the three compared populations, except for rs11694064 and rs11695186 whose minor allele frequencies are similar in the European and African populations but are almost zero in the Asian population, according to the data collected from 1000 Genomes Project (Table 5). Although we detected three different haplotypes in the melanoma cell lines and primary melanocytes studied, a total of six different haplotypes have been reported worldwide in the 1000 Genomes database, whose genealogical relationship suggests the existence of two different main lineages (Figure 6). This fact typically occurs under certain non-neutral conditions such as under balancing selection. Tajima’s *D* test suggests that this could actually be the case, which adds evolutionary relevance to the diversity patterns of *ILKAP*. We ignore which functional mechanism is shaping *ILKAP* diversity but it is likely relevant for the survival of the species and adds meaning to the association of rs6431588 to melanoma risk.

In summary, we have found that *ANXA5* and *ILKAP* expression are down-regulated at the transcriptional level in MM cells compared to melanocytes, suggesting that these two genes could have an important role in MM. Moreover, we have detected two SNPs associated with MM in these genes for the first time: rs6854854 on the *ANXA5* gene and rs6431588 on the *ILKAP* gene. Both SNPs show different allele frequencies among populations that differ in geographical location and additionally *ILKAP* region is under balancing selection, which suggest the role of the environment in MM susceptibility.

## Materials and Methods

### SNPs Genotyping

#### Ethics statement

All subjects gave written informed consent and the study was approved by the Ethics Committee of Cruces and Basurto Universitary Hospitals (Bizkaia, Spain); Gregorio Marañón Hospital (Madrid, Spain) and University Clinic Hospital (Valencia,Spain).

#### Genotyping study participants

A total number of 1,883 patients with melanoma and 1,358 cancer-free controls were genotyped in three phases. ***Discovery Phase I*** **:** A total of 464 patients with malignant melanoma (MM) were recruited from March 2004 to March 2010 at the Dermatology Services of two hospitals from the Basque Country: 246 from Basurto University Hospital (Bilbao, Spain) and 218 from Cruces University Hospital (Barakaldo, Spain). Similarly, 400 cancer-free controls from the Basque Country were recruited. The melanoma patients and cancer-free controls in this study were of Caucasian origin based on their self-declared ethnicity and the overall demographics of the region. Written informed consents were obtained from all the study participants and the study was approved by both Hospital Ethics Committees. ***Validation Phase II*** **:** The second phase of the study, consisting of an independent validation series, was composed of 507 patients with melanoma recruited from the Dermatology Services of three Spanish hospitals: 211 from Gregorio Marañón General University Hospital (Madrid, Spain), 188 from La Paz University Hospital (Madrid, Spain) and 108 from Ramón y Cajal University Hospital (Madrid, Spain). A total of 383 cancer-free controls were recruited from the same geographical region (Madrid, Spain). ***Validation Phase III*** **:** The third phase of the study was composed of 912 patients with melanoma recruited from the Dermatology Services of five different hospitals: 92 from Basurto University Hospital (Bilbao, Spain), 122 from Castellon Province Hospital (Castellón, Spain), 207 from Hospital Dr. Negrin from Las Palmas (Gran Canaria, Spain), 166 from Gregorio Marañón General University Hospital (Madrid, Spain), and 373 from Instituto Valenciano de Oncología (Valencia, Spain). Similarly, 581 cancer-free controls were recruited from the geographical regions covered by the hospitals involved in this third phase of the study.

#### SNP selection and genotyping

Proteomic analyses were performed on six melanoma cell lines (A375, Hs294T, HT-144, 1205Lu, WM793B, JSG) and four primary melanocytes (HEMn-LP, HEMn-MP, HEMn-DP, HEMa-LP). On the other hand, Affymetrix Human U133A GeneChip arrays were used to analyze and compare mRNA expression profiles on four melanoma cell lines isolated from patients’ biopsies in our laboratory (which were named JEM, JSG1, JSG2, MJOI) and three benign nevi from patients’ skin lesions isolated also in our laboratory (named FDR, JPA, RRR).

By combining the information obtained from the results of protein analysis by two-dimensional gel electrophoresis (2-D PAGE), Western blot and mRNA expression microarrays performed previously by our group (data no shown), we produced a list of candidate genes related to melanoma and selected the potential SNPs using the program Haploview (www.broadinstitute.org/haploview) and HapMap Phase 1 & 2 full dataset (http://hapmap.ncbi.nlm.nih.gov/). Finally, a total of 384 SNPs located in 88 different genes were chosen related with cancer for their involvement in cell growth, cell cycle and apoptosis, cell signaling, transcription and stress response.

In Phase I, SNPs were genotyped using the GoldenGate Genotyping Assay system according to the manufacturer’s protocol (Illumina, San Diego, CA, USA) using services from Progenika Biopharma, Bizkaia, Spain. Genotyping was carried out using 350 ng of DNA per reaction and genotypes were called using the proprietary software supplied by Illumina (BeadStudio, v3.1.3.). In validation phases, 15 SNPs (rs6854854, *ANXA5*; rs13167522 and rs1882619, *APC*; rs4135385, *CTNNB1*; rs4874163, *EEF1D*; rs7997328, *GAS2*; rs6431588, *ILKAP*; rs7212835, *PSMD11*; rs17800727, *RBL2*; rs11719889, *RUVBL1*; rs2241715, *TGFB1*; rs3733868 and rs6556466, *TTC1*; rs655539, *TXNL1*; and rs11042431, *WEE1*) were genotyped using the KASPAR SNP Genotyping System (KBiosciences, Hoddesdon, UK). The PCR was carried out according to the manufacturer’s instructions. The genotype of each sample was determined by measuring allele-specific final fluorescence in an ABI Prism 7900HT Detection System, using the SDS 2.3 software for allele discrimination (Applied Biosystems, Foster City, CA, USA). As a quality control measure, one sample duplicate and a non-template sample per 96-well plate were included.

#### Genotyping statistical analysis

For all polymorphisms studied in Phase I, Fisher’s exact test was used to account for differences in allele frequencies between HapMap CEU data and population data from the Basque Country, to test for deviations from Hardy-Weinberg equilibrium (HWE) among controls, and to compare allele counts between cases and controls. Correction for multiple testing was carried out using the Bonferroni method based on a final set of 314 accepted marker loci. Genotype-related odds ratios, their corresponding 95% confidence intervals and associated p-*values* were estimated via logistic regression using SPSS v.17 and the online software “Hardy-Weinberg equilibrium” (http://ihg2.helmholtz-muenchen.de/cgi-bin/hw/hwa1.pl). Statistical analysis of the SNPs located in chromosome X was done with the genotypes of women only. Genotype frequencies were also compared between MM cases and controls using the Cochran-Armitage trend test.

### ILKAP Expression and Sequencing in Melanomas and Primary Melanocytes

#### Cell lines

In the present work, ten melanoma cell lines and three primary melanocytes were used. The three primary human melanocytes were purchased from Invitrogen (Cat. No. C-002-5C for lightly pigmented neonatal foreskin, HEMn-LP; Cat. No. C-102-5C for moderately pigmented neonatal foreskin, HEMn-MP; and Cat. No. C-202-5C for darkly pigmented adult foreskin, HEMn-DP). All primary human melanocytes were grown in Cascade Medium 254 supplemented with Cascade Human Melanocyte Growth Supplement (both from Invitrogen; Carlsbad, CA, USA) in the absence of antibiotics.

Likewise, we cultivated ten different melanoma cell lines. Of these, A375 (ATCC CRL-1619), Hs294T (ATCC HTB-140), HT-144 (ATCC HTB-63), WM793B (ATCC CRL-2806), and 1205Lu (ATCC CRL-2812) were purchased from American Type Culture Collection (Rockville, MD, USA); RPMI7951 (ACC76), COLO-800 (ACC193), MEL-HO (ACC62), and MEL-Juso (ACC74) were obtained from Innoprot (Derio, Bizkaia, Spain); and JSG was established and characterized in our laboratory from a surgical primary melanoma as described previously [39]. The melanoma cell lines were cultured in appropriate medium supplemented with 10% fetal bovine serum (FBS), 2 mM L-glutamin and antibiotics according to the manufacturer’s instruction. All primary human melanocytes and melanoma cell lines were cultured at 37°C with 5% CO_2_ and 95% humidity.

#### Gene expression by quantitative real-time PCR (RT-qPCR)

Total RNA was isolated from ten melanoma cell lines (A375, Hs294T, HT-144, 1205Lu, WM793B, JSG, MEL-HO, MEL-Juso, COLO-800, RPMI7951) and three primary melanocytes (HEMn-LP, HEMn-MP, HEMn-DP) using the RNeasy Mini kit (Qiagen Inc., Hilden, Germany). For each sample, cDNA was synthesized from 1 µg total RNA using the iScript™ cDNA Synthesis kit (Bio-Rad Laboratories, Hercules, CA, USA) according to the manufacturer’s instructions. Real-time RT-PCR assays were carried out using an iCycler PCR platform (Bio-Rad Laboratories, Hercules, CA, USA). The reaction mixture contained 0.1 µl cDNA from the reverse transcription reaction, together with forward and reverse specific primers and iQ™ SYBR® Green Supermix (Bio-Rad Laboratories, Hercules, CA, USA) in a final reaction volume of 20 µl. The PCR reaction began by heating at 95°C for 10min, followed by 45 cycles of denaturation at 95°C for 30s, annealing at the corresponding temperature for each gene (56–61°C) for 20s and extension at 72°C for 30s. Each assay included a negative control with no template. Expression data were generated from 2 amplification reactions with samples and controls run in triplicate. Optical data obtained by real-time PCR were analyzed using the MyiQ Single-Color Real-Time PCR Detection System Software, Version 1.0 (Bio-Rad Laboratories, Hercules, CA, USA). Melt Curve analysis of each PCR assay and 1.5% agarose gel electrophoresis analysis of randomly selected samples were performed to confirm the specificity of the amplification products. To normalize expression data obtained from the studied genes, we used the expression of three different housekeeping genes (*ACTB*, *GAPDH*, and *RPS15*) and the Gene Expression Macro Software v.1.1 (Bio-Rad Laboratories, Hercules, CA, USA), where the relative expression values were computed by the comparative Ct method [40], [41]. The sequences of primers used were: *ANXA5*, forward 5′-CAGCGGATGTTGGTGGTTC-3′ and reverse 5′-CAGCCTGAAATAAAGCCTGAG-3′; *ILKAP*, forward 5′-AAGTTTGTAAAGCCTCTTCGGTG-3′ and reverse 5′-CTCGGTGATGTCGTTCAGGAG-3′; *ACTB*, forward 5′-AGATGACCCAGATCATGTTTGAG-3′ and reverse 5′-GTCACCGGAGTCCATCACG-3′; *GADPH*, forward 5′-CCTGTTCGACAGTCAGCCG-3′ and reverse 5′-CGACCAAATCCGTTGACTCC-3′; *RPS15*, forward 5′-TTCCGCAAGTTCACCTACC-3′ and reverse 5′-CGGGCCGGCCATGCTTTACG-3′.

#### *ILKAP* sequencing

*ILKAP* was sequenced in ten melanoma cell lines (A375, Hs294T, HT-144, 1205Lu, WM793B, JSG, MEL-HO, MEL-Juso, COLO-800, RPMI7951) and three primary melanocytes (HEMn-LP, HEMn-MP, HEMn-DP). DNA was amplified by PCR with specific primers and using the reaction mix ImmoMix™ Red (Gentaur, Kampenhout, Belgium) according to the following protocol: a 10min denaturation at 95°C, 35 three-step cycles (95°C for 30s, 56–60°C for 30s, and 72°C for 1min), and 10min at 72°C in an iCycler PCR platform (Bio-Rad Laboratories, Hercules, CA, USA). The removal of the unincorporated deoxynucleotide triphosphates and primers was performed using High Pure PCR Product Purification kit (Roche Molecular Biochemicals, Madrid, Spain). The purified DNA and 3.2 pmol of either the forward or reverse primer were used in standard cycle sequencing reactions with an ABI PRISM BigDye Terminator kit and run on an ABI PRISM 310 genetic analyzer (both PE Applied Biosystems, Foster City, CA). The analysis and alignment of sequences were performed using Chromas software and the BioEdit Sequence alignment Editor and the reference sequence from UCSC Genome Browser website, GRCh37/hg19 (http://www.genome.ucsc.edu/) and NCBI dbSNP 138 (http://www.ncbi.nlm.nih.gov/snp/). The sequences of primers used for *ILKAP* promoter region sequencing were: first pair, forward 5′-TCTTTGTCTCCCCATCAACC-3′ and reverse 5′-ATTCTGGCCAATTTCGATCA-3′; second pair, forward 5′-TTCCAACCCTGCAATAAACG-3′ and reverse 5′-TTCTGGAGCTCTTGCCATCT-3′. The sequences of primers used for the sequencing of *ILKAP* exons were: forward 5′-TGAGTGTCTGTCGCTGCTG-3′ and reverse 5′-AAGTCAATACCATGCGTGC-3′. Genomic DNA was used for *ILKAP* promoter region sequencing, while cDNA was used for the sequencing of *ILKAP* exons.

#### Tajima’s *D* test

The variation of nucleotide patterns from the neutral expectation was tested by the Tajima’s *D* test using DnaSP v.5.10.1. Gene diversity is controlled by the parameter theta (θ = 4 *N*_e_ µ, where *N*_e_ is the effective population size and µ the per generation mutation rate). Several sample- based estimators of theta (θ) exist, all based on the site-frequency spectrum of the mutations (SFS), that is, the distribution of the proportion of sites where the mutant is at frequency *x*. Tajima’s *D* test compares θ_k_ and θ_π_ asking about the occurrence of rare and common variants [42]. This test takes into account the number of nucleotide positions at which a polymorphism is found or, equivalently, the number of segregating sites, *k*, and the average per nucleotide diversity, *π*. Using some mathematical expressions, if the nucleotide sequence variation among our haplotypes is neutral and the population from which we sampled is in equilibrium with respect to drift and mutation, then Tajima’s *D* test should be indistinguishable from zero. If it is either negative or positive, we can infer that there’s some departure from the assumptions of neutrality and/or equilibrium.

A region of 66 Kb (chr2∶239,066,043–239,132,324 according to UCSC Genome Browser website, GRCh37/hg19), including the promoter and coding region of *ILKAP* gene, was analyzed and compared among European, Asian and African populations from 1000 Genomes Project. Standard coalescent simulations, as implemented in DnaSP, were used to estimate the statistical significance of the *D* values.

#### Western blot

Melanoma cells (A375, Hs294T, HT-144, 1205Lu, WM793B, JSG, MEL-HO, MEL-Juso, COLO-800, RPMI7951) and primary melanocytes (HEMn-LP, HEMn-MP, HEMn-DP) were harvested by trypsinization, washed with PBS and lysed in RIPA lysis buffer (80 mM Tris-HCl pH 8, 150 mM NaCl, 1% NP 40, 0.5% sodium deoxycholate, 0.1% SDS) containing Protease Inhibitor Cocktail (Sigma-Aldrich Quimica, S.A., Madrid, Spain) for 15 minutes on ice. Lysates were then cleared by centrifugation at 10,000 g for 5 minutes and total protein concentration was determined. Fifty micrograms of total proteins from each sample were resolved by electrophoresis on an SDS-polyacrylamide gel and then transferred onto a nitrocellulose membrane (Whatman GmbH, Dassel, Germany). The blots were incubated with PBS containing 5% non-fat milk and 0.1% Tween-20 for 1 hour to block nonspecific binding, and then incubated with an appropriate dilution of primary antibody at 4°C for overnight. The primary antibodies used were anti-human Annexin A5 (ab54775) and γ-tubulin (ab11320) antibodies (Abcam, Inc, Cambridge, CA). After washing, membranes were incubated for 1 hour with horseradish peroxidase-linked secondary antibody. Finally, proteins were visualized by enhanced chemiluminescence using the SuperSignal® West Pico Chemiluminescent Substrate (Thermo Scientific, Rockford, IL, USA) and the intensity of each band was measured using ImageJ software.

## Supporting Information

File S1 **Table S1.** SNPs removed in the discovery Phase I of the genotyping study. **Table S2.** List of 314 successfully genotyped SNPs, HapMap_CEU MAF, Spanish MAF, and HWE p-*value*. **Table S3.** Minor allele frequency in different populations for the 8 SNPs appearing as outliers in Figure 1 (Data from 1000 Genomes Phase I May 2011).
(DOC)
