# Supplementary material for: Involvement of ANXA5 and ILKAP in Susceptibility to Malignant Melanoma
Source: PLoS One. 2014 Apr 17;9(4):e95522. doi: 10.1371/journal.pone.0095522 (PMC3990692; doi:10.1371/journal.pone.0095522)
Supplement: File S1 — Table S1. SNPs removed in the discovery Phase I of the genotyping study. Table S2. List of 314 successfully genotyped SNPs, HapMap_CEU MAF, Spanish MAF, and HWE p-value. Table S3. Minor allele frequency in different populations for the 8 SNPs appearing as outliers in Figure 1 (Data from 1000 Genomes Phase I May 2011). (DOC) [file pone.0095522.s001.doc]

**Table S1. SNPs removed in the discovery Phase I of the genotyping study.**

| **SNPs removed** | **Reason** |
| --- | --- |
| rs2431508 in *APC*; rs3027254 in *AURKB*; rs1009316 in *BAX*; rs3181305 in *CASP14*; rs1563148 in *EEF1D*; rs1640233 in *FSCN1*; rs11842990 in *GAS6*; rs7770 in *GDI2*; rs2091767 and rs3770472 in *ITGA5*; rs396302 in *MAPKAPK3*; rs11134697 in *NPM1*; rs17354559 in *PIAS3*; rs3193024 in *RCC2*; rs11606329 in *RELA*; rs1543442 in *SNAI1*; rs13306708 in *TGFB1* | Genotyped in less than 85% of the samples |
| rs10113 in *CALM3*; rs1138272 in *GSTP1*; rs375171 in *HDAC5*; rs3770472 in *IGFBP5*; rs10972558 in *TPM2* | Low quality genotyping |
| rs4705693, rs6875894 and rs459552 in *APC*; rs7249222 in *AXL*; rs11554376 in *CDK2*; rs5743390 in *CTNNB1*; rs7992146 in *GAS6*; rs6675747 in *HDGF*; rs11615567 in *ITGA5*; rs9724642 in *NRAS*; rs13335284 in *PDPK1*; rs12040700 in *PEA15*; rs1983954 in *PIAS3*; rs6672511 in *RCC2*; rs7312221 in *SP1*; rs7163020 in *THBS1*; rs12520927 in *TTC1*; rs2234521 in *WARS* | Monomorphic |
| rs17718 in *ANXA5*; rs6594650 and rs377860 in *APC*; rs1004955 in *AXL*; rs2231426 in *CCT7*; rs1045435 and rs2069413 in *CDK2*; rs3731255 in *CDKN2A*; rs1722851 and rs4135384 in *CTNNB1*; rs399427 in *HDAC5*; rs2241194 in *IGFBP5*; rs6759854 in *ILKAP*; rs9888879 in *ITGAM*; rs2170840 in *MAPKAPK3*; rs6119993 in *MAPRE1*; rs2737827 in *MCL1*; rs9724644 in *NRAS*; rs2335111 in *PDPK1*; rs1803942 in *PEA15*; rs1050057 in *PIAS3*; rs836552 in *RAC1*; rs6892604, rs17772565 and rs1047380 in *RAD50*; rs223071 in *RCN1*; rs5746129 in *SOD2*; rs2228262 in *THBS1*; rs11042429 and rs4370932 in WEE1 | Out of HWE |

HWE. Hardy-Weinberg equilibrium

**Table S2. List of 314 successfully genotyped SNPs, HapMap_CEU MAF, Spanish MAF, and HWE p-*value*.**

| **Gene** | **Chrom** | **SNP** | **HapMap_CEU minor allele** | **HapMap_CEU MAF** | **Spanish MAF** | **HWE p-*value*** |
| --- | --- | --- | --- | --- | --- | --- |
| *ANXA5* | 4 | rs2306419 | A | 0.084 | 0.077 | 1 |
| *ANXA5* | 4 | rs6857766 | A | 0.239 | 0.196 | 0.8960 |
| *ANXA5* | 4 | rs2306421 | G | 0.236 | 0.298 | 0.1479 |
| *ANXA5* | 4 | rs6854854 | C | 0.110 | 0.115 | 0.0027 |
| *ANXA5* | 4 | rs6823422 | G | 0.475 | 0.481 | 0.0202 |
| *ANXA5* | 4 | rs17449954 | C | 0.066 | 0.080 | 0.0017 |
| *ANXA5* | 4 | rs2306416 | C | 0.159 | 0.129 | 0.8569 |
| *APC* | 5 | rs2229995 | A | 0.032 | 0.016 | 0.1737 |
| *APC* | 5 | rs1882619 | C | 0.071 | 0.083 | 0.0615 |
| *APC* | 5 | rs2707761 | G | 0.125 | 0.138 | 0.1257 |
| *APC* | 5 | rs411356 | C | 0.341 | 0.348 | 0.0575 |
| *APC* | 5 | rs454886 | C | 0.358 | 0.345 | 0.7760 |
| *APC* | 5 | rs4987109 | C | 0 | 0.009 | 0.0004 |
| *APC* | 5 | rs2431507 | G | 0.107 | 0.095 | 1 |
| *APC* | 5 | rs13167522 | C | 0.091 | 0.125 | 0.4210 |
| *APC* | 5 | rs2909786 | A | 0.458 | 0.427 | 1 |
| *AR* | X | rs12014709 | G | 0.111 | 0.101 | 0.0821 |
| *AR* | X | rs9332968 | C | 0 | 0.015 | 0.0082 |
| *AR* | X | rs5919394 | C | 0.178 | 0.163 | 0.3611 |
| *AR* | X | rs9332970 | C | 0 | 0.008 | 0.0002 |
| *AR* | X | rs5031002 | A | 0.044 | 0.029 | 1 |
| *AR* | X | rs2361634 | G | 0.075 | 0.054 | 0.0014 |
| *ARID5A* | 2 | rs2278563 | T | 0.252 | 0.272 | 0.1182 |
| *ARID5A* | 2 | rs6576973 | G | 0.017 | 0.050 | 0.0002 |
| *ARID5A* | 2 | rs749027 | A | 0.385 | 0.366 | 0.8567 |
| *AURKB* | 17 | rs2289590 | C | 0.388 | 0.451 | 0.1717 |
| *AXL* | 19 | rs1051008 | T | 0.004 | 0.011 | 1 |
| *AXL* | 19 | rs2304234 | G | 0.359 | 0.438 | 1 |
| *AXL* | 19 | rs2271546 | A | 0.119 | 0.106 | 0.8566 |
| *BAG2* | 6 | rs9370567 | C | 0.080 | 0.093 | 0.0160 |
| *BAG2* | 6 | rs9885757 | T | 0.207 | 0.271 | 0.4308 |
| *BAX* | 19 | rs4645900 | T | 0.031 | 0.039 | 1 |
| *BAX* | 19 | rs4645887 | A | 0.354 | 0.388 | 0.3943 |
| *BCL2L11* | 2 | rs2241842 | C | 0.475 | 0.434 | 0.6247 |
| *CALM3* | 19 | rs710889 | A | 0.376 | 0.392 | 1 |
| *CALM3* | 19 | rs4380146 | G | 0.345 | 0.331 | 0.4663 |
| *CASP14* | 19 | rs3181163 | C | 0.150 | 0.197 | 0.1699 |
| *CCT7* | 2 | rs12464589 | A | 0.050 | 0.050 | 0.7694 |
| *CCT7* | 2 | rs2231427 | G | 0.009 | 0.009 | 0.0004 |
| *CCT7* | 2 | rs1053329 | A | 0.321 | 0.261 | 0.4909 |
| *CCT7* | 2 | rs7557055 | A | 0.496 | 0.441 | 0.5522 |
| *CCT7* | 2 | rs523355 | C | 0.067 | 0.114 | 0.7069 |
| *CDK2* | 12 | rs2069414 | A | 0.053 | 0.050 | 1 |
| *CDK2* | 12 | rs2069408 | G | 0.310 | 0.376 | 0.3647 |
| *CDK2* | 12 | rs3213122 | A | 0.091 | 0.093 | 0.0167 |
| *CDK4* | 12 | rs2270777 | A | 0.403 | 0.472 | 0.0180 |
| *CDK4* | 12 | rs2069511 | A | 0 | 0.015 | 0.1430 |
| *CDK4* | 12 | rs2069502 | A | 0.330 | 0.220 | 0.9127 |
| *CDKN1A* | 6 | rs3176352 | G | 0.242 | 0.294 | 0.7900 |
| *CDKN1A* | 6 | rs11515 | G | 0.125 | 0.190 | 0.3713 |
| *CDKN1A* | 6 | rs2518719 | G | 0.150 | 0.169 | 0.6977 |
| *CDKN1A* | 6 | rs2811708 | T | 0.208 | 0.312 | 0.5893 |
| *CDKN1A* | 6 | rs3731239 | C | 0.412 | 0.322 | 0.9419 |
| *CLIC1* | 6 | rs3131383 | A | 0.100 | 0.067 | 0.4367 |
| *CREBBP* | 16 | rs3025702 | G | 0 | 0.023 | 0.0008 |
| *CSNK1G2* | 19 | rs4807189 | C | 0.434 | 0.405 | 0.3723 |
| *CTBP1* | 4 | rs2101094 | T | 0.155 | 0.126 | 0.5891 |
| *CTBP1* | 4 | rs900021 | T | 0.157 | 0.167 | 0.2206 |
| *CTBP1* | 4 | rs1250108 | C | 0.438 | 0.405 | 0.5914 |
| *CTBP1* | 4 | rs1265922 | A | 0.367 | 0.316 | 0.9077 |
| *CTBP1* | 4 | rs3755930 | G | 0.235 | 0.272 | 0.0967 |
| *CTNNB1* | 3 | rs11564447 | G | 0.049 | 0.072 | 0.0015 |
| *CTNNB1* | 3 | rs11564445 | T | 0.053 | 0.018 | 1 |
| *CTNNB1* | 3 | rs4135389 | A | 0 | 0.005 | 0.0163 |
| *CTNNB1* | 3 | rs2953 | T | 0.482 | 0.407 | 0.6225 |
| *CTNNB1* | 3 | rs4135385 | G | 0.283 | 0.273 | 0.7809 |
| *CTSD* | 11 | rs2292963 | T | 0.204 | 0.237 | 0.4318 |
| *DUSP1* | 5 | rs2431663 | G | 0.044 | 0.049 | 0.4190 |
| *EEF1D* | 8 | rs4874163 | G | 0.208 | 0.156 | 0.2535 |
| *EEF1D* | 8 | rs3793367 | G | 0.117 | 0.166 | 0.3200 |
| *EEF1D* | 8 | rs1809148 | T | 0.146 | 0.086 | 0.4721 |
| *ENO1* | 1 | rs4908519 | T | 0.483 | 0.432 | 0.0597 |
| *ENO1* | 1 | rs3820037 | G | 0.161 | 0.181 | 0.8351 |
| *ENO1* | 1 | rs2781066 | A | 0.190 | 0.151 | 0.4327 |
| *ENO1* | 1 | rs11544514 | A | 0 | 0.008 | 0.0397 |
| *ENO1* | 1 | rs2781064 | G | 0.465 | 0.429 | 0.1862 |
| *ENO1* | 1 | rs11811795 | T | 0.150 | 0.160 | 0.7175 |
| *FASTK* | 7 | rs2288648 | A | 0.004 | 0.008 | 1 |
| *FASTK* | 7 | rs2303942 | G | 0.495 | 0.418 | 0.3161 |
| *FOS* | 14 | rs7101 | C | 0.257 | 0.282 | 0.2282 |
| *FOS* | 14 | rs1063169 | T | 0.152 | 0.103 | 0.8305 |
| *FSCN1* | 7 | rs852479 | T | 0.052 | 0.057 | 0.6136 |
| *FSCN1* | 7 | rs1640234 | G | 0.111 | 0.106 | 1 |
| *FSCN1* | 7 | rs3801004 | G | 0.113 | 0.132 | 0.1574 |
| *GAS6* | 13 | rs9577922 | C | 0.195 | 0.220 | 0.0603 |
| *GAS6* | 13 | rs8191973 | G | 0.110 | 0.109 | 0.4956 |
| *GAS6* | 13 | rs7399637 | A | 0.367 | 0.411 | 0.6772 |
| *GAS6* | 13 | rs9604573 | T | 0.241 | 0.280 | 0.9209 |
| *GAS6* | 13 | rs11842558 | C | 0.044 | 0.087 | 0.0940 |
| *GAS6* | 13 | rs7399860 | C | 0.181 | 0.137 | 0.0383 |
| *GAS6* | 13 | rs6602910 | G | 0.376 | 0.390 | 0.3709 |
| *GAS6* | 13 | rs7997328 | C | 0.283 | 0.269 | 0.2017 |
| *GDI2* | 10 | rs12570881 | T | 0.173 | 0.207 | 0.8446 |
| *GDI2* | 10 | rs2497 | C | 0.375 | 0.465 | 0.4214 |
| *GDI2* | 10 | rs1858446 | C | 0.252 | 0.284 | 1 |
| *GDI2* | 10 | rs1129614 | T | 0.208 | 0.195 | 0.0556 |
| *GDI2* | 10 | rs4748916 | C | 0.354 | 0.417 | 0.1056 |
| *GDI2* | 10 | rs1567705 | G | 0.115 | 0.115 | 0.3828 |
| *GPX1* | 3 | rs1800668 | T | 0.233 | 0.338 | 0.1046 |
| *GRB2* | 17 | rs16967789 | A | 0.127 | 0.109 | 0.5367 |
| *GRB2* | 17 | rs7219 | G | 0.212 | 0.271 | 0.0167 |
| *GRB2* | 17 | rs4789172 | T | 0.475 | 0.487 | 0.8666 |
| *GRB2* | 17 | rs4788891 | A | 0.142 | 0.198 | 0.3161 |
| *GRB2* | 17 | rs4350602 | C | 0.250 | 0.288 | 0.0657 |
| *GSTP1* | 11 | rs4147581 | C | 0.491 | 0,.461 | 0.3282 |
| *GSTP1* | 11 | rs4986949 | T | 0 | 0.004 | 1 |
| *GSTP1* | 11 | rs749174 | T | 0.407 | 0.321 | 1 |
| *HDAC5* | 17 | rs11079983 | T | 0.297 | 0.275 | 0.6726 |
| *HDAC5* | 17 | rs8065686 | T | 0.093 | 0.114 | 0.9221 |
| *HDAC5* | 17 | rs575798 | T | 0.292 | 0.292 | 0.9974 |
| *HDGF* | 1 | rs11264533 | C | 0.288 | 0.352 | 0.2602 |
| *HDGF* | 1 | rs6682846 | T | 0.226 | 0.211 | 0.1316 |
| *HTATIP* | 11 | rs3372 | A | 0.350 | 0.400 | 0.7486 |
| *HTATIP* | 11 | rs4645933 | T | 0.348 | 0.279 | 0.3522 |
| *HTATIP* | 11 | rs487264 | T | 0.097 | 0.097 | 0.2248 |
| *HTATIP* | 11 | rs1151500 | T | 0.097 | 0.106 | 0.3713 |
| *IGFBP5* | 2 | rs11575194 | T | 0.040 | 0.057 | 0.5743 |
| *IGFBP5* | 2 | rs741384 | G | 0.450 | 0.469 | 0.4356 |
| *IGFBP5* | 2 | rs11575134 | A | 0.190 | 0.222 | 0.6882 |
| *IGFBP5* | 2 | rs3276 | A | 0.053 | 0.032 | 0.6322 |
| *IGFBP5* | 2 | rs7426116 | G | 0.342 | 0.352 | 0.6385 |
| *IGFBP5* | 2 | rs2241193 | A | 0.124 | 0.153 | 0.2087 |
| *IGFBP5* | 2 | rs3755137 | T | 0.225 | 0.208 | 0.2032 |
| *ILKAP* | 2 | rs10385 | T | 0 | 0.006 | 0.0167 |
| *ILKAP* | 2 | rs7604350 | A | 0.145 | 0.236 | 0.6931 |
| *ILKAP* | 2 | rs6722284 | A | 0.059 | 0.041 | 0.0379 |
| *ILKAP* | 2 | rs7558403 | T | 0.333 | 0.313 | 1 |
| *ILKAP* | 2 | rs6431588 | T | 0.175 | 0.146 | 0.8952 |
| *ILKAP* | 2 | rs2880131 | G | 0.204 | 0.233 | 0.5245 |
| *ILKAP* | 2 | rs2305171 | T | 0.146 | 0.161 | 0.5091 |
| *IMPDH2* | 3 | rs11706052 | G | 0.115 | 0.114 | 0.3495 |
| *ITGA5* | 12 | rs1270919 | G | 0.133 | 0.104 | 0.0199 |
| *ITGA5* | 12 | rs12318746 | A | 0.013 | 0.011 | 0.0007 |
| *ITGAM* | 16 | rs11861251 | C | 0.142 | 0.138 | 0.4475 |
| *ITGAM* | 16 | rs9937837 | G | 0.230 | 0.306 | 0.7891 |
| *ITGAM* | 16 | rs2359661 | T | 0.434 | 0.459 | 0.0131 |
| *ITGAM* | 16 | rs4077810 | T | 0.327 | 0.283 | 0.3036 |
| *ITGAM* | 16 | rs8048583 | T | 0.358 | 0.291 | 0.4428 |
| *ITGAM* | 16 | rs7193268 | T | 0.128 | 0.223 | 0.8943 |
| *ITGAM* | 16 | rs11645653 | C | 0.243 | 0.267 | 0.0088 |
| *ITGAM* | 16 | rs4597342 | T | 0.327 | 0.280 | 0.6891 |
| *JUN* | 1 | rs11688 | A | 0.058 | 0.063 | 0.0024 |
| *MAGED1* | X | rs12689461 | A | 0.005 | 0.015 | 0.0002 |
| *MAGED1* | X | rs1992271 | T | 0.306 | 0.275 | 0.0780 |
| *MAGED1* | X | rs12852833 | G | 0.142 | 0.110 | 0.0006 |
| *MAGED1* | X | rs3199687 | A | 0.225 | 0.186 | 0.1059 |
| *MAPKAPK3* | 3 | rs9879397 | A | 0.009 | 0.021 | 0.3007 |
| *MAPKAPK3* | 3 | rs11711534 | A | 0.063 | 0.085 | 0.9812 |
| *MAPKAPK3* | 3 | rs2040397 | A | 0.252 | 0.278 | 0.3957 |
| *MAPKAPK3* | 3 | rs11130254 | G | 0.133 | 0.148 | 0.0300 |
| *MAPRE1* | 20 | rs7271735 | C | 0.093 | 0.073 | 0.0034 |
| *MAPRE1* | 20 | rs13045669 | G | 0.065 | 0.039 | 0.0021 |
| *MAPRE1* | 20 | rs17123673 | G | 0.066 | 0.043 | 0.2746 |
| *MAPRE1* | 20 | rs2235760 | T | 0.059 | 0.149 | 0.8936 |
| *MAPRE1* | 20 | rs242553 | T | 0.467 | 0.497 | 0.6622 |
| *MAPRE1* | 20 | rs20654 | G | 0.279 | 0.252 | 0.0557 |
| *MCL1* | 1 | rs12036617 | T | 0 | 0.007 | 0.0265 |
| *MCL1* | 1 | rs878471 | G | 0.417 | 0.486 | 1 |
| *MYD88* | 3 | rs4988457 | G | 0.058 | 0.030 | 0.0051 |
| *MYD88* | 3 | rs7744 | G | 0.146 | 0.155 | 0.4776 |
| *MYD88* | 3 | rs6853 | G | 0.115 | 0.130 | 1 |
| *MYD88* | 3 | rs989298 | A | 0 | 0.007 | 0.0264 |
| *NDN* | 15 | rs1722807 | A | 0 | 0.011 | 0.0008 |
| *NME2* | 17 | rs4605213 | C | 0.407 | 0.361 | 0.0271 |
| *NPM1* | 5 | rs12189191 | T | 0.319 | 0.391 | 0.2270 |
| *NPM1* | 5 | rs17748299 | G | 0.044 | 0.041 | 1 |
| *NPM1* | 5 | rs7711765 | T | 0.353 | 0.423 | 0.2934 |
| *NRAS* | 1 | rs14804 | T | 0.250 | 0.216 | 0.3280 |
| *PARK7* | 1 | rs4908488 | C | 0.129 | 0.136 | 0.0441 |
| *PARK7* | 1 | rs226256 | C | 0.417 | 0.449 | 0.8204 |
| *PARK7* | 1 | rs3766606 | A | 0.168 | 0.165 | 1 |
| *PARK7* | 1 | rs178932 | G | 0.375 | 0.342 | 0.7076 |
| *PDPK1* | 16 | rs1005273 | T | 0.412 | 0.458 | 0.0244 |
| *PEA15* | 1 | rs680083 | A | 0.403 | 0.438 | 0.8342 |
| *PEA15* | 1 | rs16831591 | G | 0.044 | 0.044 | 0.0066 |
| *PEBP1* | 12 | rs2088702 | C | 0.327 | 0.440 | 0.2832 |
| *PEBP1* | 12 | rs2936840 | T | 0.323 | 0.322 | 0.0588 |
| *PIM1* | 6 | rs9349029 | T | 0 | 0.007 | 0.0273 |
| *PIM1* | 6 | rs10507 | T | 0.327 | 0.267 | 0.4361 |
| *PIR* | X | rs234502 | C | 0.134 | 0.093 | 0.0017 |
| *PIR* | X | rs6632532 | T | 0.045 | 0.028 | 1 |
| *PIR* | X | rs5935980 | A | 0.346 | 0.237 | 0.5049 |
| *PIR* | X | rs7052755 | A | 0.371 | 0.438 | 0.8274 |
| *PIR* | X | rs1996173 | T | 0 | 0.016 | 0.0005 |
| *PIR* | X | rs2271550 | T | 0.356 | 0.344 | 0.6843 |
| *PIR* | X | rs1983287 | A | 0.318 | 0.299 | 0.3180 |
| *PIR* | X | rs2071177 | T | 0.331 | 0.499 | 0.6473 |
| *PIR* | X | rs7064724 | G | 0.353 | 0.409 | 1 |
| *PIR* | X | rs4830945 | A | 0.491 | 0.444 | 0.3329 |
| *PIR* | X | rs1003026 | T | 0.243 | 0.314 | 0.4690 |
| *PIR* | X | rs5935959 | A | 0.219 | 0.198 | 0.7183 |
| *PIR* | X | rs4830946 | C | 0.345 | 0.302 | 0.3149 |
| *PIR* | X | rs432654 | A | 0.062 | 0.111 | 0.2123 |
| *PKM2* | 15 | rs8192402 | A | 0.051 | 0.075 | 0.2774 |
| *PKM2* | 15 | rs2959910 | C | 0 | 0.009 | 0.0004 |
| *PKM2* | 15 | rs2856929 | G | 0.195 | 0.185 | 0.6676 |
| *PKM2* | 15 | rs2052713 | C | 0.229 | 0.255 | 0.5429 |
| *PRDX1* | 1 | rs11211129 | T | 0.469 | 0.459 | 0.9972 |
| *PRDX1* | 1 | rs2152077 | G | 0.314 | 0.327 | 0.4279 |
| *PRDX1* | 1 | rs2065708 | G | 0.212 | 0.219 | 0.3567 |
| *PRDX3* | 10 | rs3740562 | A | 0.279 | 0.334 | 0.9101 |
| *PRDX3* | 10 | rs11554902 | A | 0 | 0.005 | 0.0160 |
| *PSMB3* | 17 | rs764190 | T | 0.075 | 0.055 | 0.1817 |
| *PSMB3* | 17 | rs228274 | A | 0.150 | 0.164 | 1 |
| *PSMB3* | 17 | rs65926 | C | 0.212 | 0.187 | 0.0131 |
| *PSMB3* | 17 | rs228275 | T | 0.425 | 0.341 | 0.4786 |
| *PSMD11* | 17 | rs7212835 | G | 0.128 | 0.165 | 0.3986 |
| *PSMD11* | 17 | rs9900089 | C | 0.376 | 0.409 | 0.9178 |
| *PSMD11* | 17 | rs12162135 | T | 0.447 | 0.472 | 0.4360 |
| *RAC1* | 7 | rs6951997 | G | 0.009 | 0.040 | 0.0032 |
| *RAC1* | 7 | rs702484 | C | 0.279 | 0.281 | 0.9272 |
| *RAC1* | 7 | rs12977 | A | 0 | 0.056 | 0.1829 |
| *RAC1* | 7 | rs836547 | T | 0.071 | 0.101 | 0.9111 |
| *RAC1* | 7 | rs6967221 | A | 0.058 | 0.057 | 0.6763 |
| *RAC1* | 7 | rs12536544 | A | 0.250 | 0.266 | 0.3861 |
| *RAC1* | 7 | rs836551 | G | 0.475 | 0.499 | 0.2875 |
| *RAD50* | 5 | rs1047380 | G | 0 | 0.017 | 0.0790 |
| *RAD50* | 5 | rs2237060 | C | 0.375 | 0.404 | 0.3170 |
| *RAD50* | 5 | rs4526098 | G | 0 | 0.030 | 0.0049 |
| *RAD50* | 5 | rs7449456 | T | 0.202 | 0.209 | 0.1005 |
| *RAP1B* | 12 | rs10506562 | A | 0.207 | 0.210 | 0.1245 |
| *RAP1B* | 12 | rs2431657 | A | 0.119 | 0.139 | 0.0649 |
| *RAP1B* | 12 | rs11177321 | A | 0.115 | 0.094 | 0.0518 |
| *RAP1B* | 12 | rs11177315 | G | 0.398 | 0.328 | 0.5918 |
| *RAP1B* | 12 | rs2439759 | T | 0.226 | 0.296 | 0.1460 |
| *RBL2* | 16 | rs4784311 | T | 0.158 | 0.188 | 0.4434 |
| *RBL2* | 16 | rs17800727 | G | 0.254 | 0.413 | 0.0063 |
| *RBL2* | 16 | rs13329946 | T | 0.051 | 0.067 | 0.9706 |
| *RBL2* | 16 | rs3929 | C | 0.394 | 0.392 | 0.0757 |
| *RCC2* | 1 | rs6586542 | C | 0.412 | 0.276 | 0.1438 |
| *RCC2* | 1 | rs12728891 | C | 0.062 | 0.127 | 0.0402 |
| *RCC2* | 1 | rs1204897 | A | 0.168 | 0.208 | 0.9462 |
| *RCC2* | 1 | rs2489611 | T | 0.363 | 0.417 | 0.4817 |
| *RCN1* | 11 | rs2073223 | A | 0.465 | 0.420 | 0.0609 |
| *RELA* | 11 | rs11820062 | T | 0.491 | 0.444 | 0.7946 |
| *RELA* | 11 | rs7119750 | T | 0.155 | 0.143 | 0.4652 |
| *RELA* | 11 | rs11227247 | G | 0.146 | 0.151 | 0.5251 |
| *RPSA* | 3 | rs7621701 | A | 0.129 | 0.218 | 0.6884 |
| *RPSA* | 3 | rs2269349 | C | 0.438 | 0.484 | 0.4659 |
| *RPSA* | 3 | rs3772141 | C | 0.297 | 0.307 | 0.7185 |
| *RUVBL1* | 3 | rs3732402 | G | 0.354 | 0.344 | 0.2028 |
| *RUVBL1* | 3 | rs1057220 | G | 0.221 | 0.228 | 0.4002 |
| *RUVBL1* | 3 | rs11719889 | A | 0.258 | 0.220 | 0.7718 |
| *RUVBL1* | 3 | rs11720239 | G | 0.088 | 0.149 | 1 |
| *RUVBL1* | 3 | rs6788879 | A | 0.128 | 0.111 | 0.2853 |
| *RUVBL1* | 3 | rs7641133 | T | 0.217 | 0.240 | 0.2679 |
| *RUVBL1* | 3 | rs7650365 | G | 0.491 | 0.479 | 0.9522 |
| *RUVBL2* | 19 | rs1062708 | C | 0.447 | 0.457 | 0.6024 |
| *RUVBL2* | 19 | rs3764621 | A | 0.067 | 0.104 | 0.8378 |
| *RUVBL2* | 19 | rs753307 | T | 0.442 | 0.495 | 0.7713 |
| *RUVBL2* | 19 | rs12610125 | A | 0.067 | 0.062 | 1 |
| *SIAH2* | 3 | rs7622742 | T | 0 | 0.003 | 1 |
| *SIAH2* | 3 | rs8072 | T | 0.004 | 0.014 | 0.1201 |
| *SIAH2* | 3 | rs1148369 | A | 0.261 | 0.230 | 0.4079 |
| *SIAH2* | 3 | rs11717897 | A | 0.076 | 0.076 | 1 |
| *SNAI1* | 20 | rs4647958 | C | 0.117 | 0.126 | 0.6834 |
| *SNAI1* | 20 | rs1047920 | T | 0.079 | 0.062 | 0.3076 |
| *SNAI1* | 20 | rs6012791 | C | 0.175 | 0.186 | 1 |
| *SOD2* | 6 | rs5746136 | A | 0.327 | 0.323 | 0.1388 |
| *SOD2* | 6 | rs5746151 | A | 0.080 | 0.074 | 1 |
| *SOD2* | 6 | rs4987023 | A | 0 | 0.004 | 0.0080 |
| *SOD2* | 6 | rs2758331 | A | 0.438 | 0.446 | 1 |
| *SP1* | 12 | rs12368491 | A | 0.164 | 0.156 | 0.0330 |
| *SP1* | 12 | rs17695156 | T | 0.067 | 0.072 | 0.8517 |
| *SP1* | 12 | rs7134628 | A | 0.089 | 0.097 | 1 |
| *SPRR2G* | 1 | rs509194 | C | 0.425 | 0.430 | 0.3534 |
| *SPRR2G* | 1 | rs510277 | C | 0.425 | 0.427 | 0.4104 |
| *TFDP1* | 13 | rs2316121 | A | 0.425 | 0,.421 | 0.9835 |
| *TGFB1* | 19 | rs11466338 | G | 0.066 | 0.090 | 0.3441 |
| *TGFB1* | 19 | rs2241715 | T | 0.283 | 0.370 | 0.8408 |
| *TGFB1* | 19 | rs4803455 | A | 0.496 | 0.466 | 0.9500 |
| *TGFB1* | 19 | rs8110090 | G | 0.045 | 0.069 | 0.0107 |
| *THBS1* | 15 | rs2292305 | C | 0.097 | 0.150 | 0.3378 |
| *THBS1* | 15 | rs17633107 | C | 0.117 | 0.198 | 0.4841 |
| *THBS1* | 15 | rs3743125 | T | 0.106 | 0.137 | 0.4151 |
| *THBS1* | 15 | rs1478604 | G | 0.243 | 0.347 | 0.6798 |
| *THBS1* | 15 | rs1051442 | T | 0.119 | 0.182 | 1 |
| *TPM2* | 9 | rs17259350 | T | 0.106 | 0.077 | 1 |
| *TPM2* | 9 | rs1243872 | G | 0.473 | 0.470 | 0.8392 |
| *TPM2* | 9 | rs3793537 | C | 0.250 | 0.276 | 0.9560 |
| *TPM2* | 9 | rs1998308 | A | 0.372 | 0.354 | 0.3797 |
| *TTC1* | 5 | rs3733868 | T | 0.073 | 0.068 | 0.9728 |
| *TTC1* | 5 | rs3733869 | T | 0.319 | 0.338 | 0.8832 |
| *TTC1* | 5 | rs6556466 | C | 0.066 | 0.094 | 0.0185 |
| *TTC1* | 5 | rs11749533 | A | 0.066 | 0.119 | 1 |
| *TTC1* | 5 | rs4921106 | G | 0.153 | 0.199 | 0.0116 |
| *TTC1* | 5 | rs10515804 | T | 0.367 | 0.306 | 0.0838 |
| *TTC1* | 5 | rs7704770 | G | 0.381 | 0.439 | 0.0348 |
| *TXNL1* | 18 | rs681176 | A | 0.133 | 0.168 | 0.6586 |
| *TXNL1* | 18 | rs655539 | C | 0.049 | 0.106 | 0.0854 |
| *TXNL1* | 18 | rs10513898 | T | 0.075 | 0.051 | 0.4777 |
| *TXNL1* | 18 | rs2286984 | T | 0.254 | 0.250 | 0.8380 |
| *UBE2L6* | 11 | rs2729371 | T | 0.221 | 0.182 | 0.8137 |
| *UBE2L6* | 11 | rs2848624 | A | 0.434 | 0.396 | 0.0310 |
| *UBE2L6* | 11 | rs5847 | T | 0.438 | 0.496 | 0.8957 |
| *WARS* | 14 | rs2234519 | A | 0.013 | 0.029 | 1 |
| *WARS* | 14 | rs12882639 | T | 0.475 | 0.481 | 0.0974 |
| *WARS* | 14 | rs7155068 | T | 0.242 | 0.251 | 0.8782 |
| *WARS* | 14 | rs724392 | A | 0.252 | 0.270 | 0.3184 |
| *WEE1* | 11 | rs11042431 | G | 0.150 | 0.211 | 0.0034 |
| *WNT5A* | 3 | rs10865994 | A | 0.454 | 0.409 | 0.1326 |
| *WNT5A* | 3 | rs3773608 | C | 0.106 | 0.112 | 0.9943 |
| *WNT5A* | 3 | rs7622120 | A | 0.478 | 0.401 | 0.1288 |
| *WNT5A* | 3 | rs1829556 | G | 0.465 | 0.420 | 0.2458 |
| *WNT5A* | 3 | rs648872 | T | 0.162 | 0.184 | 0.9826 |
| *WNT5A* | 3 | rs566926 | A | 0.270 | 0.222 | 0.6992 |
| *WNT5A* | 3 | rs11918967 | C | 0.442 | 0.487 | 0.2295 |
| *YWHAZ* | 8 | rs3134377 | C | 0.127 | 0.232 | 0.2865 |
| *YWHAZ* | 8 | rs3134354 | G | 0.062 | 0.046 | 1 |
| *YWHAZ* | 8 | rs17365305 | A | 0.053 | 0.044 | 0.9769 |
| *YWHAZ* | 8 | rs17462921 | A | 0.174 | 0.186 | 0.7822 |
| *YWHAZ* | 8 | rs17365661 | C | 0.071 | 0.074 | 0.0186 |
| *YWHAZ* | 8 | rs3134358 | G | 0.389 | 0.407 | 0.1515 |
| *YWHAZ* | 8 | rs4734497 | C | 0.366 | 0.295 | 0.3250 |

Chrom. Chromosome; MAF. Minor Allele Frequency; HWE. Hardy-Weinberg Equilibrium.

**Table S3. Minor allele frequency in different populations for the 8 SNPs appearing as outliers in Figure 1 (Data from 1000 Genomes Phase I May 2011).**

|  |  | **Europe (n=380)** | | **Africa (n=246)** | | **Asia (n=286)** | |
| --- | --- | --- | --- | --- | --- | --- | --- |
| **SNP** | **Gene** | **MiA** | **MAF** | **MiA** | **MAF** | **MiA** | **MAF** |
| rs2069502 | *CDK4* | T | 0.326 | T | 0.057 | C | 0.320 |
| rs3731239 | *CDKN1A* | G | 0.386 | G | 0.012 | G | 0.114 |
| rs2303942 | *FASTK* | A | 0.475 | G | 0.311 | A | 0.439 |
| rs2497 | *GDI2* | G | 0.351 | G | 0.246 | G | 0.371 |
| rs2088702 | *PEBP1* | C | 0.388 | T | 0.297 | T | 0.402 |
| rs228275 | *PSMB3* | T | 0.368 | T | 0.157 | C | 0.343 |
| rs17800727 | *RBL2* | G | 0.279 | G | 0.028 | G | 0.002 |
| rs6586542 | *RCC2* | C | 0.371 | G | 0.496 | C | 0.173 |

MiA. Minor allele; MAF. Minor Allele Frequency.
